# Comparing effect latencies in the visual world paradigm: Monte Carlo simulations to assess resampling-based procedures

**DOI:** 10.3758/s13428-025-02934-6

**Published:** 2026-02-23

**Authors:** Serge Minor

**Affiliations:** UiT – The Arctic University of Norway, Stakkevollan, PO Box 6050, N-9037 Tromsø, Norway

**Keywords:** Visual world eye tracking, Latency analysis, Divergence point analysis, Monte Carlo simulations, Bootstrapping, Permutation

## Abstract

In a series of Monte Carlo simulation studies, we evaluated the power and Type I error rates of resampling-based procedures for comparing effect latencies between groups in the visual world paradigm (VWP). Resampling-based methods, while versatile, are known to fail in certain cases. Therefore, validation of such methods through simulation is crucial. We compared permutation- and bootstrapping-based tests combined with different methods for measuring effect latency while manipulating sample size and true effect size. Alongside previously used latency measures, we tested new measures involving the application of an effect size threshold. Simulations were based on existing VWP datasets representing different effect types (preferential looks triggered by lexical vs. grammatical cues, cohort competitor effects in word recognition) and data collection methods (infrared- vs. webcam-based eye tracking). A total of 156,000 simulations were conducted across five studies, involving 548 million resampled datasets. The main findings are as follows: (1) With sufficient sample sizes, tests were effective in detecting latency differences of 200–300 ms in sentence processing tasks, and as small as 100 ms in word recognition. (2) The permutation test and bootstrapped percentile CIs exhibited the highest overall power without inflation of Type I error rates. (3) Applying an effect size threshold in latency estimation led to consistent increases in statistical power. (4) Resampling by participant was robust to increases in cross-subject variability;in contrast, bootstrapping within participants and time bins led to elevated Type I error rates. Based on these results, we offer recommendations for using non-parametric resampling-based procedures to compare group latencies in VWP experiments.

**Supplementary Information:**

The online version contains supplementary material available at 10.3758/s13428-025-02934-6.

## Introduction

### The visual world paradigm

Since the pioneering work of Cooper (1974), and especially over the last three decades following Tanenhaus et al. (1995), eye tracking in the visual world paradigm (VWP) has been a valuable tool in language processing research. In VWP experiments, participants’ eye movements are recorded in response to visual stimuli (pictures or words on a screen or objects in semi-realistic scenes) during the presentation of spoken language input. Although details of the linking hypothesis for the VWP remain a matter of debate (see Salverda & Tanenhaus, 2017; Magnuson, 2019), existing results suggest that listeners’ eye movements closely track important aspects of linguistic processing. Thanks to its high temporal resolution, the VWP has provided insight into real-time processing of syntactic ambiguity (Tanenhaus et al., 1995; Trueswell et al., 1999), online prediction based on lexical and grammatical cues and world knowledge (Altman and Kamide, 1999, 2007; Kamide et al., 2003a, b; Lew-Williams & Farnald, 2007, 2010), the timing of pragmatic inferences (Huang and Snedeker, 2009), fine-grained dynamics of word recognition (Allopenna et al., 1998), and more (see Huettig et al., 2011, for an overview).

**Fig. 1:**
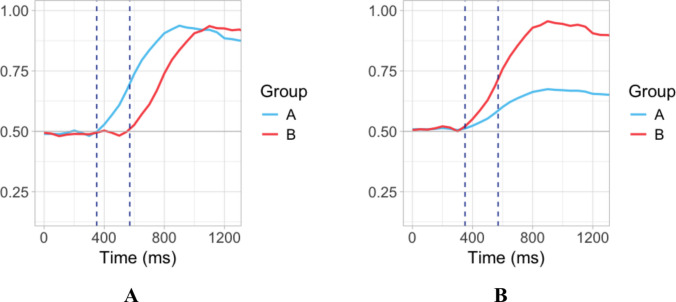
Simulated data illustrating proportions of looks to target area of interest (AOI) in a two-picture visual world design for two groups of participants. **A** Groups differ in effect onset. Vertical lines represent time window boundaries where a difference in effect size reflects a difference in effect onset. **B** Groups differ in effect size but not in effect onset

In a typical VWP experiment, researchers compare looks to particular pictures or objects in a scene (areas of interest, or AOIs) while manipulating some aspect of the linguistic stimulus. Usually, the aim of the analysis is to compare the strength of preference for particular AOIs as reflected in the number/length of fixations within a predetermined time window of interest (see Ito & Knoeferle, 2023, for a recent overview of analysis methods in the VWP). However, it is often also of theoretical interest to determine *when an effect starts*, e.g., when the participants first begin to exhibit a preference for a particular picture/object, and to compare the onsets of the effect between groups or conditions. In some cases, this question can be addressed by comparing the proportions of looks to the target item between the groups/conditions. However, this method implies that the researcher must know in advance the critical time window where a difference in fixation proportions may be interpreted as a difference in effect timing—typically, a narrow time window after the effect first obtains in the faster group/condition (Fig. 1A). And even if it is possible to select such a time window in advance, a difference in the *size of the effect* does not necessarily correspond to a difference in* effect timing* (Fig. 1B).

### Effect onset analysis in the VWP

Stone et al. (2020) proposed a procedure to address the question of effect timing in the VWP more directly by using non-parametric bootstrapping to estimate the uncertainty around effect onsets, and to statistically assess the difference in effect onsets between groups. Bootstrapping involves resampling the experimental data many times with replacement, which makes it possible to use the experimental sample to estimate the sampling distribution of a statistic of interest, such as a difference in effect onsets (Efron & Tibshirani, 1993; Manly, 2006). Stone et al.’s procedure consists of three steps:

1. *Calculating the difference in effect latencies*. Effect latency for each group is established by dividing the broad time window of interest into smaller time bins (e.g., of 20 or 50 ms) and conducting a statistical test for the effect of interest in each time bin (e.g., by applying a *t*-test or linear model). Then the first of several (e.g., five) consecutive time bins with a significant effect is taken as the measure of effect latency (see also Borovsky et al., 2012; Ito et al., 2018). The latency in one group is then subtracted from the latency in the other group to obtain a difference in effect latencies between the groups.
2. *Performing a non-parametric bootstrap.* A non-parametric bootstrap is performed, which involves resampling the data with replacement 1,000–2,000 times (Efron & Tibshirani, 1993). On each resampling iteration, effect latencies in both groups and their difference are calculated using the same method as for the original dataset (described in step 1).
3. *Calculating a bootstrapped confidence interval.* Resampling the data many times generates a bootstrap distribution of the effect latencies and their differences. Uncertainty around these estimates can then be quantified by calculating confidence intervals (CIs). If the 95% CI for the difference in effect latencies does not include 0, this can be taken as evidence that the effect began earlier in one group than the other.

An alternative to the bootstrapping approach is to perform a non-parametric permutation test to establish whether the difference in effect latencies between the groups is statistically significant. The permutation test addresses the likelihood that the observed difference in latencies between two groups will occur if the null hypothesis is in fact true, i.e., if belonging to one group or the other does not impact the effect latency (Good, 1994). A permutation-based procedure for comparing effect latencies in the VWP would proceed as follows (see, e.g., Minor et al., 2022):

1. *Calculating the difference in effect latencies*. This step is the same as in the bootstrapping approach.
2. *Performing random permutations.* The group labels in the original dataset are randomly permuted, and the difference in effect latencies is re-computed in the same way as for the original dataset. This is repeated many (e.g., 1,000) times.
3. *Calculating the p-value*. Multiple random permutations of the data produce a distribution of the difference in effect latencies *under the null hypothesis* that the group labels are interchangeable. We can then calculate a *p*-value for the observed difference in effect latencies as the proportion of values in the null hypothesis distribution which are at least as extreme as the observed value. If this proportion is below 0.05, we have grounds to reject the null hypothesis and conclude that the observed difference between the groups is significant.

Stone et al. (2020) applied the bootstrapping technique to compare how quickly L1 and L2 speakers of German are able to use grammatical gender information to identify the target referent of a noun phrase (see also Stone et al., 2021, for a related study). The participants saw four objects depicted on the screen and heard instructions containing a noun phrase describing the target object, where both the determiner and the adjective were marked for grammatical gender, e.g., “*Click on the*_*masc*_*blue*_*masc*_*button*.” Out of the four pictures on the screen, one was the target, matching both the gender encoded on the determiner and adjective and the color described by the adjective. Out of the remaining three pictures, one shared the same color as the target but differed in gender (= color competitor), one shared the same gender but differed in color (= gender competitor), and finally one differed in both color and gender from the target (= distractor). Stone et al. (2020) compared the times when the participants in the L1 and L2 groups exhibited more looks to the target picture than to the color competitor. Prior to the presentation of the noun, such a preference would indicate that the participants were able to use the grammatical gender information on the determiner and adjective to anticipate the target object. The bootstrapped 95% confidence interval for the difference in effect latencies between the L1 and L2 groups was [160, 340] ms (mean 244 ms), indicating that L1 speakers were significantly quicker than L2 speakers in identifying the target referent. Moreover, the 95% CI for the effect onset preceded the onset of the noun in the L1 group but not in the L2 group, suggesting that L1 speakers were relying on grammatical gender cues to identify the target.

Minor et al. (2022) used a combination of bootstrapping and permutation-based procedures to compare the onsets of target preference for different types of grammatical aspect marking in Russian. The authors presented Russian-speaking participants with contrasting pictures of ongoing and completed events, and spoken sentences involving verbs marked with a perfective or imperfective grammatical aspect (see also Zhou et al., 2014). In Russian, depending on the verb, grammatical aspect information is marked either by the presence/absence of a prefix on the verb or by the presence/absence of a suffix. Applying the bootstrapping and permutation methods, Minor et al. found that the participants were able to identify the target picture (ongoing events for the imperfective aspect and completed events for the perfective) significantly earlier when the aspectual information was marked by a prefix than when it was marked by a suffix (*p = *0.008, bootstrapped mean difference between the conditions, 210 ms; 95% CI [58, 358] ms).

### Infrared versus webcam-based eye tracking

The majority of VWP eye-tracking studies to date have been conducted using specially designed eye-tracking devices that combine an infrared light source and an infrared camera. Such eye trackers capture gaze direction with high precision and at a high and constant sampling rate, typically 100–1,000 Hz. Recently, however, a new method of collecting eye-tracking data has been introduced that makes use of standard computer webcams to automatically estimate gaze direction (Papoutsaki et al., 2017; Valliappan et al., 2020; Park et al., 2020). The main advantage of this method is that it does not require specialized equipment, making it possible to collect eye-tracking data via the web, which greatly facilitates participant recruitment.

Most existing webcam-based eye tracking studies have employed WebGazer—a free and open-source JavaScript application which analyzes eye images captured by standard consumer webcams and calculates gaze directions in the form of *x,y* screen coordinates (Papoutsaki et al., 2016). Several recent studies have shown that language processing effects previously found with the help of infrared eye trackers can be reproduced using webcam-based eye tracking with WebGazer (Degen et al., 2021; Yang & Krjabich, 2021; Vos et al., 2022; Slim & Hartsuiker, 2023; Prystauka et al., 2024). In a webcam-based online study, Vos et al. (2022) were able to closely replicate the pattern of looks triggered by the processing of aspectual verb forms in English originally reported in Minor et al. (2023). Slim and Hartsuiker (2023) used webcam-based eye tracking and WebGazer to replicate the effect of verbal semantics on target preference, finding more looks to the target picture after constraining verbs compared to a neutral condition (Dijkgraaf et al., 2017; Altmann & Kamide, 1999). Prystauka et al. (2024) reproduced this effect again in an online webcam-based study, and were also able to replicate a subtler effect of lexical interference from adjective semantics, originally reported in Kukona et al. (2014). With regard to latency analysis, in a recent Webgazer-based VW study, Vanek et al. (2024) used bootstrap CIs to compare the timing of looks during the processing of negative sentences with simple negation and negative quantifiers in Croatian and English, but found no significant differences between the conditions.

At the same time, studies have reported lower spatial and temporal precision for data collected using WebGazer than for infrared eye-tracking data (Semmelmann & Weigelt, 2018; Degen et al., 2021; Slim & Hartsuiker, 2023). Moreover, data collected with WebGazer are generally characterized by higher variability due to differences in the participants’ hardware and surrounding conditions during the experiment (e.g., webcam quality, computer processing capacity, lighting conditions). For instance, lab-based infrared eye trackers collect gaze samples at a high sampling rate, which is kept constant for all the participants (e.g., 60 or 120 Hz). The sampling rate in webcam-based eye tracking, on the other hand, is influenced by the hardware specifications of each participant’s computer, and typically varies anywhere between 1 and 50 Hz (Semmelmann & Weigelt, 2018; Vos et al., 2022; Prystauka et al., 2024).

Nevertheless, webcam-based eye tracking has been gaining in popularity in recent years, especially following the outbreak of the COVID-19 pandemic. WebGazer has been integrated into several popular tools for conducting web-based behavioral experiments, including Gorilla (Anwyl-Irvine et al., 2020), jsPsych (de Leeuw, 2015), and PCIbex (Zehr & Schwarz, 2018). For this reason, we decided to include webcam-based eye-tracking data in the current study, with the dual goal of (a) testing the robustness of statistical procedures for effect latency analysis when applied to sparser and noisier webcam-based data, and at the same time (b) assessing the viability of webcam-based eye tracking, as a relatively novel methodology, to study effect latency differences in the VWP.

### Simulation-based assessment of latency comparison procedures

While resampling-based methods are generally flexible and have been shown to be applicable to a wide range of common statistics, they are also known to fail in certain cases (see Chernick & LaBudde, 2011, for detailed discussion). For instance, a well-known scenario where bootstrapping fails to provide a reliable estimate of sampling error is in the estimation of extreme values in a distribution (i.e., the minimum or maximum). It is not immediately clear how well these methods would perform for complex derived measures such as estimates of effect latency in the VWP; therefore, validating them through either theoretical analysis or simulation is crucial.

In a series of studies, we evaluated the power and false-positive rates of bootstrapping- and permutation-based procedures for effect latency comparison in the VWP, focusing on between-subject designs (i.e., comparisons *between groups* of participants). Given the complexity of the analysis procedures, as well as the complex structure of the analyzed VWP eye-tracking data, it is hard to assess these methods analytically. We therefore used Monte Carlo simulations to evaluate the procedures under a range of conditions. We manipulated a range of properties of the simulated datasets (type of effect of interest, data collection method, sample size, true effect size, and degree of between-subject variability) and parameters of the procedures themselves, including choice of latency measure, choice of test, and choice of resampling method.

#### Datasets

**Fig. 2:**
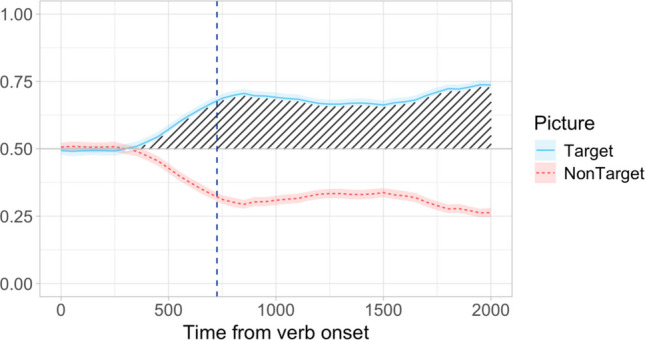
Dataset A: Proportion of looks to target and competitor pictures calculated in 50-ms time bins starting from the onset of the verb. Looks to white space have been removed. The vertical dashed blue line indicates the average verb offset (726 ms). Shading marks the effect of interest

To ensure that the simulated datasets were as realistic as possible, they were generated based on real experimental VWP eye-tracking data. Since the accuracy and reliability of the procedures may differ depending on the properties of the effect of interest, as well as methods of data collection (e.g., in-lab infrared eye tracking vs. over-the-web webcam-based eye tracking), we conducted simulation studies based on four different datasets. Dataset A was a subset of the data from Minor et al. (2022) which included all the filler trials from that study (24 items). The dataset comprised eye-tracking recordings from 124 adult Russian-speaking participants (mean age 22), with 24 trials per participant. In each filler trial, the participants saw two pictures of events involving an agent performing different actions (e.g., a boy sawing a log vs. a boy writing a letter) and heard an audio recording of a sentence describing one of these pictures. The sentence always involved a subject-verb-object structure, and the target picture could be identified as soon as the verb was presented (e.g., *Malčik ****pilil**** dlinnoje brevno* “A boy **was sawing** a long log”). The participants were asked to listen to the sentences and select the matching pictures by raising one hand (left or right). The pictures were presented on a 22-inch monitor, and the participants’ eye movements were recorded using an SMI RED 500 infrared eye tracker attached to the bottom of the monitor sampling at 120 Hz. Unsurprisingly, the participants were at ceiling in identifying the target picture, and exhibited a lasting target preference effect in the eye-tracking data. Visual inspection of the gaze plot suggests that this effect started soon after the onset of the verb in the audio stimulus (Fig. 2).

**Fig. 3:**
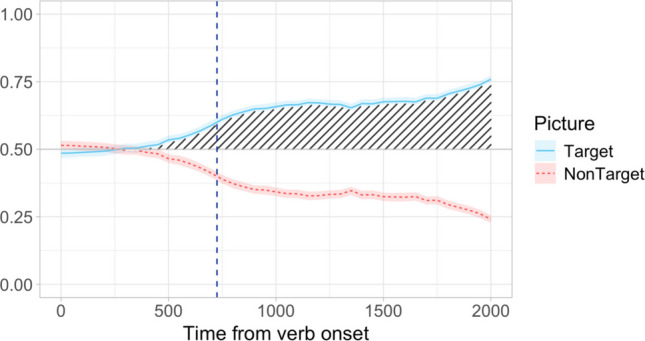
Dataset B: Proportion of looks to target and competitor pictures calculated in 50-ms time bins starting from the onset of the verb. Looks to white space have been removed. The vertical dashed blue line indicates the average verb offset (726 ms). Shading marks the effect of interest

Dataset B was a subset of the data produced in a webcam-based replication of Minor et al.’s (2022) study. The experiment was programmed in jsPsych, and eye-tracking data were collected from 240 participants over the web using Webgazer.js. Once again, Dataset B included only the filler trials from the experiment (24 items). The materials and structure of each trial were the same as in Minor et al. (2022), except that the participants selected the target picture by clicking. As in the original experiment, the participants showed a strong and early target preference effect in the eye-tracking data (Fig. 3).

Datasets A and B thus encode a lasting target preference effect in a two-picture VWP setup triggered by the processing of a lexical cue (the verb). These datasets were selected for three reasons. First, we wanted to obtain a *benchmark of efficacy* for the latency analysis procedures when applied to effects triggered by particularly salient cues with a limited amount of expected variation between speakers. Second, the relatively large samples of participants in these datasets provide a better representation of the between-subject variability that exists in the broader population than that provided by smaller samples, and allowed us to produce subsample simulations with a wide range of group sizes (12, 24, 36, 48, and 60 participants per group for Dataset A, and 12, 24, 36, 48, 60, 80, 100, and 120 participants per group for Dataset B). Finally, since Dataset B was derived from a closely matched webcam-based replication of the study that generated Dataset A, we were able to directly compare the accuracy and reliability of the latency analysis procedures when applied to infrared and webcam-based eye-tracking data. These were the goals of Studies 1 and 2.

**Fig. 4:**
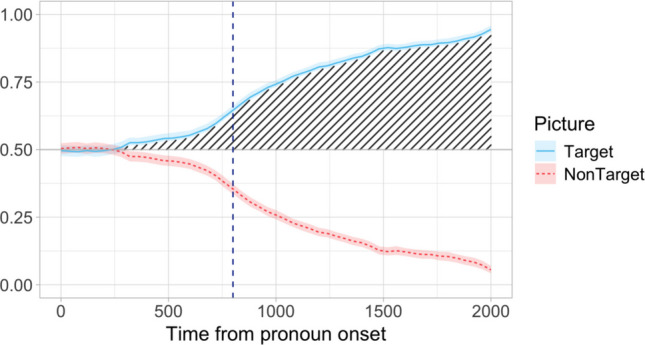
Dataset C: Proportion of looks to target and competitor pictures calculated in 40-ms time bins starting from the onset of the possessive pronoun. Looks to white space have been removed. The vertical dashed blue line indicates the onset of the following adjective (800 ms). Shading marks the effect of interest

Dataset C was a subset of the data from Stone et al. (2021)—an infrared eye-tracking study that looked at predictive use of grammatical gender cues on possessive pronouns in German. Specifically, the study tested for the existence of a mismatch effect when the antecedent gender encoded in the stem of the possessive pronoun was mismatched to the gender of the head noun encoded in an agreement inflection on the pronoun (e.g., *Klick auf seine gelbe Flasche* “Click on **his**_**.FEM**_ yellow_.FEM_ bottle_.FEM_”). The processing of such mismatches was compared to sentences where the antecedent gender and the head noun gender matched (e.g., *Klick auf ihre gelbe Flasche* “Click on **her**_**.FEM**_ yellow_.FEM_ bottle_.FEM_”). The study included two experiments, in a four-picture and a two-picture setup, each comparing a match and a mismatch condition. Dataset C comprised the data from the match condition in Experiment 2, which involved a two-picture setup with two objects matching in color but differing in grammatical gender. There were 48 items in the match condition, and data were collected from 69 adult speakers of German (*M*_age_ = 26, age range 18–53).^1^ Figure 4 illustrates target and competitor looks starting from the onset of the possessive pronoun.

Thus, the target preference effect observed in Dataset C was triggered by a different kind of cue than Datasets A and B, namely a grammatical gender cue that the participants could use to predict the upcoming noun. In Study 3, we were interested in testing the latency analysis procedures when applied to this type of effect. The relatively large sample size in Dataset C allowed us to conduct simulations with subsamples ranging from 12 to 34 participants per group.

**Fig. 5:**
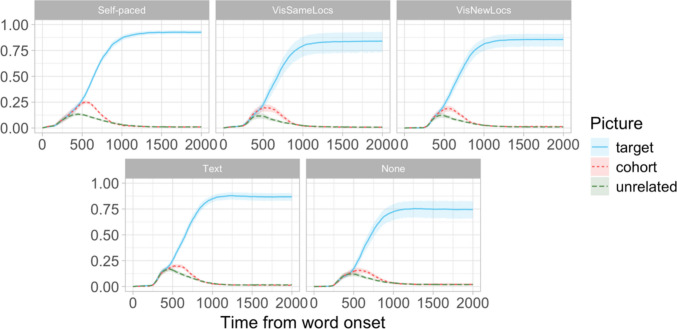
Dataset D (Apfelbaum et al., 2021): Proportions of looks to the target, cohort competitor, and unrelated objects by preview condition. Unrelated looks represent the mean proportion of fixations to the two unrelated objects

Finally, Dataset D was the shared data from Apfelbaum et al. (2021). The study used VW eye tracking to investigate lexical word recognition and the effects of picture preview duration on fixation patterns. Each trial involved four pictures displayed in the four corners of the screen: the target item, a cohort competitor which overlapped with the target item in the initial consonants and vowels but different in the final consonant (e.g., *brain* vs. *braid*), and two unrelated items. The participants heard the target word while looking at the pictures, and had to click on the correct picture. Each participant completed 192 trials. The dataset included gaze recording from 119 English L1-speaking participants who were randomly assigned to one of five conditions: no preview, text preview (the names of the objects were displayed for 1.5 s before the trial began), visual–new locations (the item pictures were displayed for 1.5 s in a diamond configuration before each trial), visual–same locations (the item pictures were previewed for 1.5 s in the same locations as in the trial), and self-paced (the pictures were displayed in the correct locations until the participants clicked on a dot to start the trial). Apfelbaum et al.’s analysis revealed a significant competitor effect (cohort vs. unrelated) in all the conditions, but the size of this effect differed between the conditions: the largest effect was observed in the self-paced and visual–same locations conditions, a smaller effect in the visual–new locations condition, and the smallest effect in the no preview and text preview conditions (Fig. 5).

**Fig. 6:**
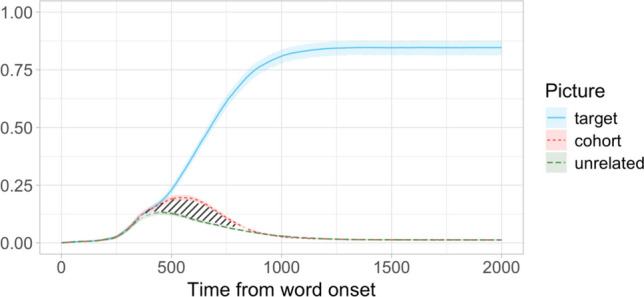
Dataset D: Proportion of looks to target, cohort competitor, and unrelated pictures across the preview conditions. Shading marks the effect of interest

In Study 4, we were interested in how the resampling-based methods of latency comparison would fare when applied to the “competitor-over-unrelated”-type effects commonly measured in word recognition studies. Such effects are different from target preference effects represented in Datasets A–C in two important respects: they are generally smaller, and they have a limited duration—whereas target preference effects tend to last, or even increase, until the end of the trial, competitor preference effects disappear once the correct target is identified. To evaluate the power and false-positive rates of the latency comparison procedures in this context, we used the whole dataset from Apfelbaum et al. (2021) across the five preview conditions, which allowed us to include data from all 119 participants (Fig. 6). To control for potential additional variation associated with the different preview conditions in the experiment, we included an equal number of participants from each condition in every Monte Carlo simulation (see below).

#### True effect size and sample size

**Fig. 7:**
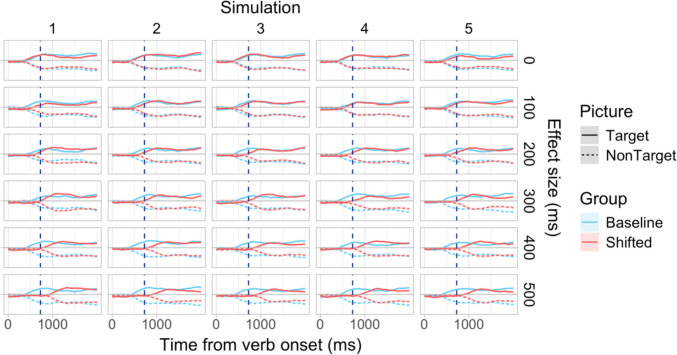
Examples of simulated data sampled from Dataset A with group size *n = *36 for a range of effect sizes (= true differences in latency between groups)

To assess the power and precision of the latency analysis procedures, we needed to generate simulated datasets with a known true effect size—in this case, a known difference in effect latencies between groups. To achieve this, we employed an approach similar to that of Kiesel et al. (2008; see also Miller et al., 1998): We began with a dataset where no a priori difference in effect latencies was expected (i.e., data generated by participants sampled from a uniform population tested in the same experimental condition), and for each simulation we randomly sampled 2 × *n* participants from this dataset without replacement. These participants were then randomly divided into two groups of size *n*, the Baseline and Shifted groups. No transformation was applied to the data in the Baseline group. For the Shifted group, a fixed amount was added to all the time indices, i.e., all the data were effectively shifted to the right on the time axis by a fixed interval. The size of this shift corresponded to the true latency difference between the Baseline and Shifted groups (see Fig. 7 for an illustration). We then applied the bootstrap- and permutation-based procedures to test for the difference in effect latencies between the groups. Thus, each simulation represented one full experiment. Simulations were conducted for latency differences of 100, 200, 300, 400, and 500 ms in Studies 1–3, and 50, 100, and 200 ms in Study 4. In order to evaluate the Type I error rates of the analysis procedures, we conducted simulations where neither the Baseline nor the Shifted group’s time indices were modified, i.e., the true effect size was 0.

In addition to manipulating the size of the effect, we manipulated the sample size of participants in each group. We ran simulations for a range of sample sizes from 12 to 120 participants per group depending on the size of the original dataset (see above). A total of 1,000 simulations were conducted for each combination of effect and sample size.

#### Choice of latency measure

We evaluated several techniques to measure effect latency within each group. The first method, following Stone et al. (2020), identifies effect latency as the initial time point in a sequence of consecutive time bins showing a significant effect. This approach aims to detect the onset of a lasting effect while minimizing the influence of spurious, short-term fluctuations in the gaze patterns.

The second method selects the first time point that meets the threshold for statistical significance while controlling for multiple comparisons by applying a family-wise error correction (Holm–Bonferroni correction; for relevant discussion see Stone et al., 2020; Minor et al., 2022). Apart from providing a latency estimate, this method established the existence of a statistically significant effect.

The third technique is new and builds on the first by introducing a minimum effect size threshold. The effect latency is taken as the first in a sequence of time bins where the effect is significant *and* the size of the effect exceeds a defined minimal threshold. The effect size threshold is selected to ensure that the latency measure captures the onset of an effect that is not only statistically significant but also theoretically and/or practically relevant (cf. the notions of *minimal important difference* [MID] in clinical and behavioral research and *region of practical equivalence* in Bayesian analysis; Crosby et al., 2003; Kruschke, 2014, 2018). We hypothesized that introducing an effect size threshold could also offer statistical advantages. By minimizing the influence of early effects driven by small subsets of participants, it could reduce sampling error in measured latencies and thereby increase test power to detect latency differences. For the simulations, we selected a threshold of 55% target preference, which was small enough to meaningfully reflect the *onset* of an effect but large enough to have an impact on latency estimation given the tested sample sizes. In practice, the choice of threshold will depend on theoretical and practical considerations pertaining to a particular study and research question.

The final latency measure is also new and combines family-wise error correction with an effect size threshold, selecting the first time point that not only meets significance after family-wise correction but also exceeds the minimum effect size threshold.

#### Choice of test

Once effect latencies are measured in each group, a test must be applied to determine whether these latencies differ. Specifically, we test the null hypothesis that the difference in effect latencies between the two groups is zero. We assessed two types of non-parametric resampling-based procedures designed to test this null hypothesis. First, on each simulation, we applied a permutation test which generated a null hypothesis distribution of the statistic of interest (in our case, the difference in effect latencies between the groups) by randomly permuting the group labels. The probability of the observed value of the statistic can then be evaluated relative to this null hypothesis distribution (Good, 2005). Second, we applied a bootstrapping procedure to estimate the uncertainty around the difference in effect latencies by resampling data within each group with replacement. The null hypothesis can be rejected if the 95% bootstrap confidence interval does not include zero (Efron & Tibshirani, 1993; Davison & Hinkley, 1997; Manly, 2007). We compared several commonly used methods for computing bootstrap CIs: percentile, bias-corrected (BC), normal, empirical (a.k.a. basic and backwards percentile), and accelerated bias-corrected (ABC; DiCiccio & Efron, 1992).^2^

#### Choice of resampling procedure

As a rule, VW eye-tracking data possess a complex hierarchical structure with dependencies between data points within trials, participants, items, etc. In this context, the validity of non-parametric resampling methods hinges on selecting an appropriate resampling procedure. In the case of permutation tests, the permutation procedure must be chosen to randomize the levels of the predictor of interest while preserving the remaining structure of the data. When comparing effect latencies between groups, this entails permuting the group labels while preserving all other dependencies within the dataset. This is accomplished by permuting labels at the participant level—meaning that, in each permutation, either all or none of a participant’s data points are reassigned to the other group. This approach ensures that clustering of data by trial, item, and participant is preserved.

**Fig. 8:**
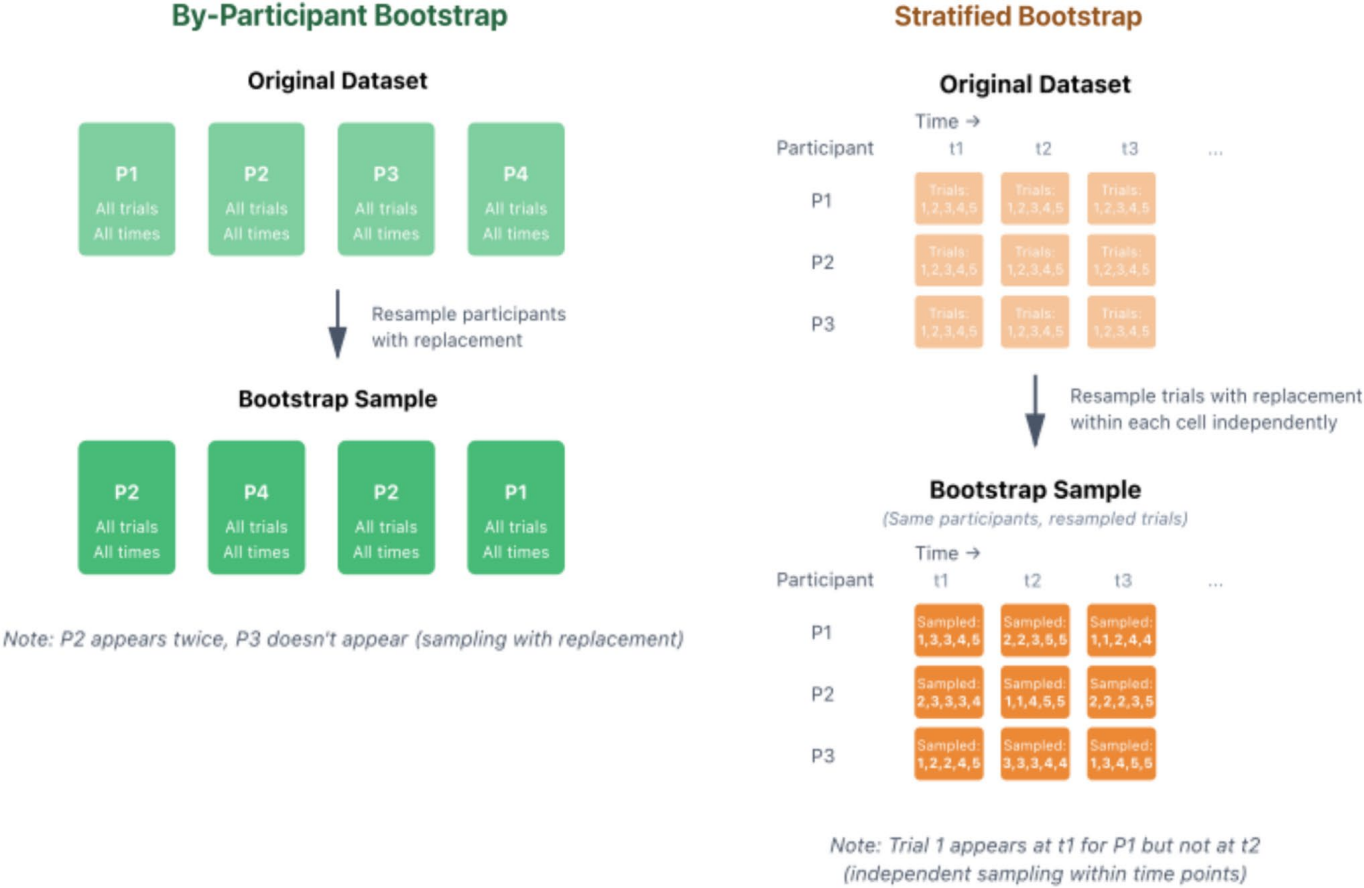
Diagrams illustrating the by-participant and stratified bootstrap procedures

A similar consideration applies in the case of the non-parametric bootstrap. The purpose of the non-parametric bootstrap procedure is to estimate the sampling distribution of a statistic, i.e., to estimate how the statistic would vary if the experiment were rerun many times with different samples. This is achieved by taking the original data sample as an estimate of the population (the so-called *plug-in principle*; Hesterberg, 2011), and resampling multiple times with replacement. Importantly, this resampling should be performed in a way that preserves the structure of the original dataset, e.g., clustering by trials, participants, and items. In most cases, this means that the resampling method should mirror the original data collection procedure (Fox, 2016). In VWP experiments, participants are typically sampled randomly (or quasi-randomly) from particular populations, and the experiments are designed to capture properties of these broader populations.^3^ Mirroring this, an adequate bootstrap procedure for group comparisons would involve resampling participants within the groups. In this case, each bootstrap resample would include two groups with the same number of participants as in the original dataset, sampled with replacement in such a way that either all or none of a participant’s data are included. We refer to this as the *by-participant bootstrap* (see diagram in Fig. 8). It ensures that the bootstrap distribution takes into account cross-subject variability while preserving existing clustering of the data. This is the procedure we adopt in Studies 1–4 (see also Minor et al., 2022).

Stone et al. (2020) propose a different bootstrap procedure for latency comparison in the VWP whereby resampling is *stratified* by participants and time points (see also Stone et al., 2021; Ito & Knoeferle, 2023; Vanek et al., 2024). With this approach, each resample is created by independently drawing samples with replacement for each participant and at each time point. For instance, if the experiment includes *n* participants with *m* trials per participant, *m* data points are randomly sampled with replacement for each participant at each time point (see diagram in Fig. 8)*.* This means that any given resampled dataset may include data from one set of trials for participant *p* at time point *i* (e.g., *p*’s gaze direction at time *i* for trials 1, 4, 4, 7, 9, etc.) and data from a different set of trials for the same participant *p* at the next time point *i* + 1 (e.g., for trials 2, 4, 5, 8, 8, etc.). Meanwhile, every resampled dataset will include data from the same set of participants as the original dataset. A drawback of this approach is that it does not preserve dependencies between data points within single trials which are known to be highly autocorrelated in VW eye-tracking data (Cho et al., 2018; Huang & Snedeker, 2020; Ito & Knoeferle, 2023). Consequently, the resampled datasets will not resemble the structure of the original data. Furthermore, because each resample is drawn from the same set of participants, the resulting bootstrap distribution may not adequately reflect cross-subject variability present in the population. This can lead to an underestimation of the uncertainty around the statistic of interest (i.e., the difference in latencies between groups) and may increase Type I error rates. We address this issue in Study 5, where we use Monte Carlo simulations to compare Type I error rates of the by-participant and stratified bootstrap procedures, and test their robustness against increased cross-subject variability.^4^

Table 1 provides a summary of all the studies conducted and the tested parameters.

**Table 1:** Summary of the studies

	Dataset	Sample sizes (per group)	Effect sizes	Latency measures	Tests	Resampling procedure(s)
Study 1	Dataset A: sentence processing, lexical cues, infrared	12, 24, 36, 48, 60	Latency differences: 100, 200, 300, 400, 500 ms	Consecutive time bins, consecutive time bins + threshold, Holm–Bonferroni-corrected, Holm–Bonferroni-corrected + threshold	Permutation, bootstrapped CIs (percentile, normal, empirical, BC, ABC)	By participant (Minor et al., 2022)
Study 2	Dataset B: sentence processing, lexical cues, web-based	12, 24, 36, 48, 60, 80, 100, 120	100, 200, 300, 400, 500 ms	*same as above*	*same as above*	By participant (Minor et al., 2022)
Study 3	Dataset C: sentence processing, grammatical cues, infrared	12, 24, 34	100, 200, 300, 400, 500 ms	*same as above*	*same as above*	By participant (Minor et al., 2022)
Study 4	Dataset D: word recognition, lexical cues, infrared	15, 25, 35, 45, 55	50, 100, 200 ms	*same as above*	*same as above*	By participant (Minor et al., 2022)
Study 5a	Dataset A	12, 24, 36, 48, 60	Random noise (*SD*): 0, 50, 100, 200 ms	*same as above*	*same as above*	By participant (Minor et al., 2022) vs. stratified by participant and time bin (Stone et al., 2020)
Study 5b	Dataset C	12, 24, 34, 48, 60	Random noise (*SD*): 0, 50, 100, 200 ms	*same as above*	*same as above*	By participant (Minor et al., 2022) vs. stratified by participant and time bin (Stone et al., 2020)

## Study 1: Infrared eye tracking, lexical cue (Dataset A)

In Study 1, we evaluated the power and reliability of permutation- and bootstrap-based procedures for detecting effect latency differences between groups when applied to VWP data collected using an infrared eye tracker. The effect of interest was target preference triggered by a lexical cue.

### Simulation protocol

We ran 1,000 simulations for each combination of group size *n* of 12, 24, 36, 48, and 60 participants per group, and *effect size* of 0, 100, 200, 300, 400, and 500 ms (30 combinations in total, 30,000 simulations). Each simulation consisted of the following steps:

Step 1. A simulated dataset was obtained by randomly sampling 2 × *n* participants without replacement from Dataset A and dividing them into two groups of *n* participants: the Baseline group and the Shifted group. The value of the effect size was added to all the time indices in the Shifted group.
Step 2. Four distinct measures of effect latency were calculated for each of the groups in the simulated dataset, as well as differences between the group latencies. For each group, we selected a 2,000-ms time region of interest (ROI) starting from the onset of the verb, i.e., the disambiguating word in the experiment. An additional 200 ms was added to the boundaries of the ROI to account for the time needed for saccade execution. All looks outside of the two pictures were removed, and proportions of looks to the target picture were calculated in forty 50-ms time bins within the ROI. A large majority of these proportions were either 0 or 1 (98.2% on average), so the data were fully binarized by replacing all proportions ≥ 0.5 with 1, and all proportions < 0.5 with 0 (cf. Huang & Snedeker, 2020; Minor et al., 2022).^5^ The following measures of the latency of target preference were obtained for each group:
  - *Consecutive time bins.* For each time bin, we used the *lme4* package in R to fit an intercept-only logistic regression model predicting the probability of looks to target. The measure of effect latency was taken as the first of five consecutive time bins where the probability of looks to target was significantly above chance (i.e., the intercept in the logistic model was significantly greater than 0).
  - *Consecutive time bins + effect size threshold.* Same as previous measure, except that the latency measure was taken as the first of five consecutive time bins where the probability of looks to target was significantly above chance *and* the size of the effect was above a predefined threshold. Specifically, the log-odds of target looks had to exceed 0.2, corresponding to 55% probability.
  - *Earliest effect after Holm–Bonferroni correction.* The latency measure was taken as the first time bin where the probability of looks to target was significantly above chance after applying the Holm–Bonferroni correction for multiple comparisons.
  - *Earliest effect after Holm–Bonferroni correction + effect size threshold.* Same as previous measure, except that the latency measure was taken as the first time bin where the probability of looks to target was significantly above chance after applying the Holm–Bonferroni correction *and* the log-odds of target looks were above 0.2 (> 55% probability).

We then calculated the difference in latencies between the Shifted and Baseline groups for each of the four latency measures.

Step 3. We conducted a *non-parametric bootstrap* for the obtained latency differences, which involved the following sub-steps:
  - Resampling *n* participants from each group with replacement 2,000 times. In each iteration, we performed the same operations as in step 2 to obtain four measures of latency difference between the groups.
  - A total of 2,000 iterations produced a bootstrap distribution for each measure. We then calculated 95% CIs for each measure of the latency difference. We tested five different methods for CI calculation: percentile, bias-corrected, normal, empirical, and accelerated bias-corrected.
Step 4. We conducted a *permutation procedure* to test the difference in effect latencies between the groups. This involved the following sub-steps:
  - We permuted the group labels by randomly re-assigning *n* participants from the simulated dataset to the Baseline group and *n* participants to the Shifted group. We then applied the procedure in step 2 to obtain latency differences between the groups in the permuted dataset. This was repeated 1000 times generating distributions of the latency differences under the null hypothesis that the true difference in latency between the groups was 0.
  - We obtained *p*-values for the latency differences in the simulated dataset relative to the null hypothesis distributions. Each *p*-value was calculated as the proportion of the null hypothesis distribution with an absolute value as large as or larger than the original estimate (two-sided test)

The numbers of bootstrap and permutation samples (2,000 and 1,000, respectively) were chosen based on the recommendations in Manly (2007) for calculating 95% bootstrap confidence intervals and conducting permutation tests at a 0.05 level of significance.

After all simulations were conducted, we evaluated the power and Type I error rate for each combination of the simulation parameters: group size, effect size, latency measure, testing procedure (bootstrap vs. permutation), and type of bootstrap CI. For the bootstrap procedure, the power to detect a nonzero true effect (under a particular combination of the other parameters) was calculated as the proportion of simulations where the lower bound of the 95% bootstrap CI was above 0. The Type I error rate was calculated based on the simulations where the true effect size was 0, and was taken as the proportion of simulations where the 95% bootstrap CI did not include 0 (i.e., the lower bound of the CI was above 0 or its upper bound was below 0). For the permutation procedure, the power to detect a nonzero true effect was taken as the proportion of simulations where the difference in effect latencies was greater than 0 with a *p*-value below 0.05. The Type I error rate was calculated as the proportion of simulations where the true effect size was 0, which produced *p*-values below 0.05.

For the bootstrap procedure, we further calculated a range of measures based on results pooled across the different effect sizes, taking a total of 6,000 simulations per group size (1,000 simulations for each of the six effect sizes). First, we calculated the overall coverage probability for each type of CI and group size as the proportion of simulations where the 95% CI of the difference in latencies included the true effect size. Next, for each type of CI and group size, we used the total results of 6,000 simulations to calculate more precise estimates of power and Type I error rate. This is possible because the *shape* of the bootstrap distribution for the latency difference obtained in each simulation does not depend on the effect size, i.e., the value that is added to the time indices in the Shifted group. Instead, the whole distribution simply shifts to the right on the time axis by the added effect size relative to effect size 0 (see Appendix B for a more detailed exposition). This means that we can add and subtract values from the estimated boundaries of the bootstrap CIs to obtain CIs for different effect sizes. In the following, we report the original estimates based on 1,000 simulations in the main text, since they allow for a more direct comparison between bootstrap CIs and the permutation test. The more precise pooled estimates for the bootstrap-based tests are given in Appendix B, and do not affect the conclusions drawn in the main text.

### Results

**Fig. 9:**
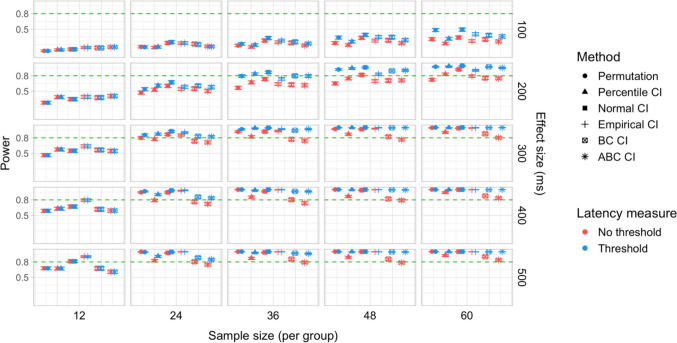
Study 1: Power estimates for analyses that calculated effect latency based on significance in consecutive time bins. Here and in the following, confidence intervals for the power estimates were calculated using the Wilson method for proportions

**Fig. 10:**
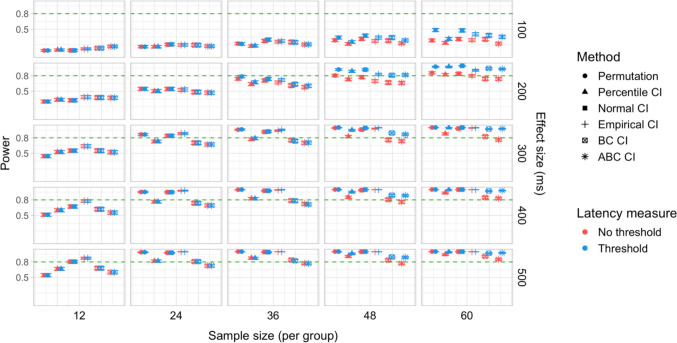
Study 1: Power estimates for analyses that calculated effect latency based on earliest effect after the application of Holm–Bonferroni correction

**Fig. 11:**
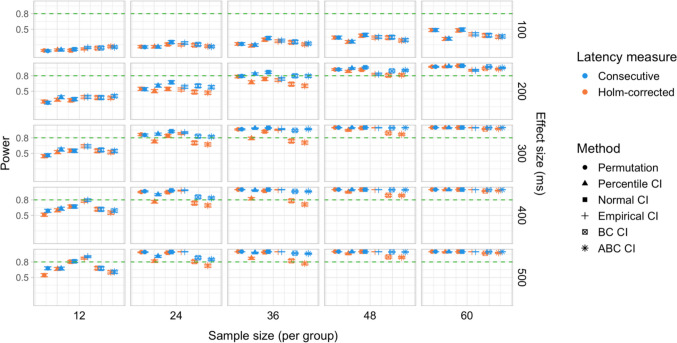
Study 1: Power comparison between latency measures based on significance in consecutive time bins and measures based on earliest effect after the application of Holm–Bonferroni correction, both with an effect size threshold

**Fig. 12:**
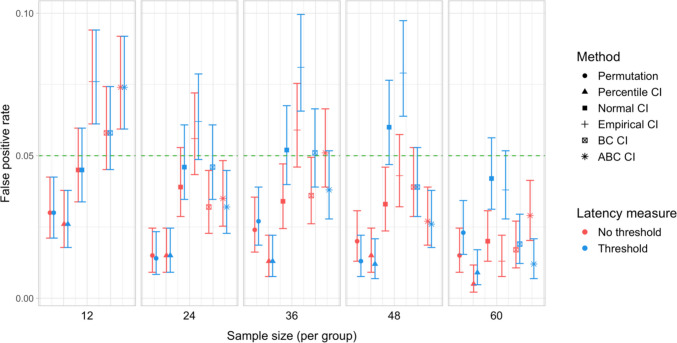
Study 1: Type I error rates for analyses that calculated effect latency based on significance in consecutive time bins

**Fig. 13:**
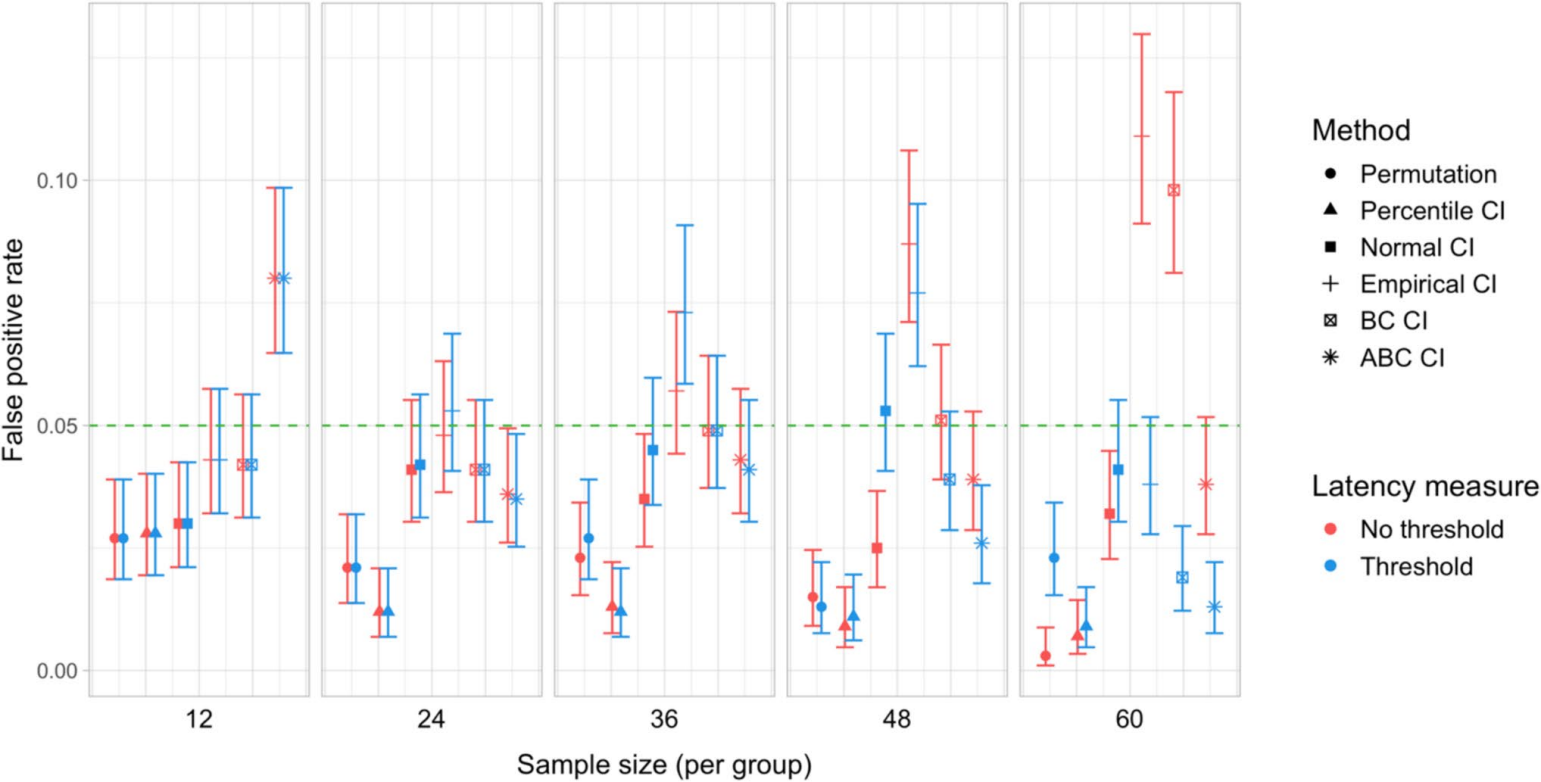
Study 1: Type I error rates for analyses that calculated effect latency based on earliest effect after the application of Holm–Bonferroni correction

**Fig. 14:**
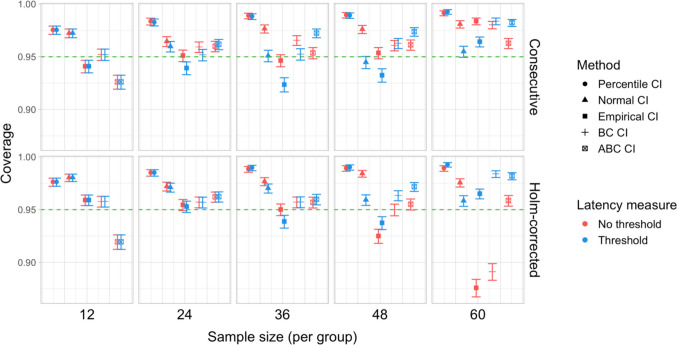
Study 1: Coverage of bootstrap confidence intervals based on data pooled across effect sizes

In total, 30,000 simulations were run, with 2,000 bootstrap resamples and 1,000 permutation resamples per simulation, amounting to 90 million resampled datasets in total for which effect latency estimates were obtained. Each latency calculation involved fitting a logistic regression model to 40 bins within the ROI for each group, which amounted to over 7.2 billion model fits. Power estimates for 600 distinct parameter combinations and Type I error rates for 120 parameter combinations were obtained. The full results are presented in Tables 1 and 2 in Appendix A, and Figs. 9, 10, 11, 12, 13, and 14.

#### Sample sizes and effect sizes

Power to detect a significant latency difference varied substantially depending on sample size and effect size (Figs. 9 and 10). For the smallest effect size (100 ms), none of the analysis procedures approached 80% power even with the largest sample size (*n = *60 per group). For 200-ms effects, multiple tests that employed an effect size threshold in latency estimation achieved 80% power for *n = *36. Without an effect size threshold, two tests achieved 80% power for *n = *48: the permutation procedure in combination with Holm–Bonferroni-based estimates (estimated power = 80.5%; 95% Wilson CI [0.78,0.83]), and bootstrap normal CIs with estimates based on consecutive time bins (estimated power = 81.7%; 95% Wilson CI [0.79, 0.84]). For effect sizes of 300, 400, and 500 ms, 80% power was achieved with multiple parameter combinations for groups of size *n = *24. For 400-ms effects, the bootstrap procedure with empirical CIs achieved 80% power already for *n = *12 (for estimates based on consecutive time bins, estimated power = 79.5%; 95% Wilson CI [0.77, 0.82]), and for the largest effect size (500 ms), bootstrap tests with empirical and normal CIs achieved 80% power for *n = *12.

#### Latency measures

We observed a consistent power advantage of applying a threshold for minimal effect size in the calculation of effect latencies, especially for small/medium latency differences (200 and 300 ms). This was the case both for latency measures based on consecutive time bins (Fig. 9), and—to a slightly lesser extent—for measures based on the application of the Holm–Bonferroni correction (Fig. 10). When the effect size threshold was applied, latency measures based on consecutive time bins slightly outperformed those that employed the Holm–Bonferroni correction in various variants of the bootstrap procedure, but the two types of measures performed equally well in the permutation procedure (Fig. 11).

#### Tests

Overall, the different methods for assessing significance performed at a comparable level. For latency measures employing an effect size threshold, the bootstrap procedure with normal CIs slightly outperformed the alternatives for several combinations of group and effect sizes, while the permutation-based procedure slightly underperformed when applied to the smallest group sizes (*n = *12).

#### Type I error rates

Figures 12 and 13 illustrate the estimated Type I error rates for all the tested parameter settings. Most variants of the procedures performed at or below the nominal level (*ɑ = *0.05). Accelerated bias-corrected (ABC) CIs significantly exceeded the nominal level when used with the smallest sample size (*n = *12). Bias-corrected CIs exceeded the nominal level in combination with the largest sample size (*n = *60) when latency measures based on the Holm–Bonferroni correction were used. Empirical CIs significantly exceeded the nominal level in multiple cases both with and without an effect size threshold.

#### Coverage

Figure 14 illustrates the overall coverage of the bootstrap CIs calculated as the proportion of simulations where 95% confidence intervals included the true effect size. The data were pooled across all effect sizes, amounting to 6,000 simulations for each combination of group size and latency measure.

Percentile and normal CIs achieved above 95% coverage for all combinations of group size and latency measure, while the ABC CIs achieved above 95% coverage for all parameter combinations with sample size larger than 12. The lowest coverage was observed for empirical CIs in several configurations, for ABC CIs when used with the smallest group sizes, and for BC CIs when used with the largest group sizes with estimates based on the Holm–Bonferroni correction.

### Discussion

We found that sample sizes of 36–48 participants per group was necessary to achieve 80% power when testing for latency differences of 200 ms; 24–36 participants per group when testing for 300-ms latency differences; and at least 24 participants per group when testing for 400–500-ms latency differences. Even 60 participants per group was not sufficient to detect latency differences of 100 ms with adequate power. A consistent increase in power was associated with the use of a 55% target preference threshold in the calculation of effect latencies.

Estimated Type I error rates for most testing procedures and parameter combinations were at or below the nominal level, although bootstrap-based tests employing empirical, bias-corrected, and accelerated bias-corrected confidence intervals significantly exceeded the nominal level for certain parameter combinations. The permutation test and percentile and normal bootstrap CIs never exceeded the 5% Type I error level. Percentile CIs also showed the highest overall coverage among the types of confidence intervals.

## Study 2: Webcam-based eye-tracking, lexical cue (Dataset B)

In the second study, we evaluated the latency analysis procedures when applied to VWP webcam-based eye-tracking data collected over the internet using the WebGazer package (Dataset B). The experiment was a replication of the infrared eye tracking study in Minor et al. (2022), which allowed for a direct comparison with the results of Study 1.

### Simulation protocol

The simulation protocol was the same as in Study 1, except that we tested a broader selection of group sample sizes (12, 24, 36, 48, 60, 80, 100, and 120 participants per group), made possible by the larger sample size in Dataset B (*n = *240).

### Results

**Fig. 15:**
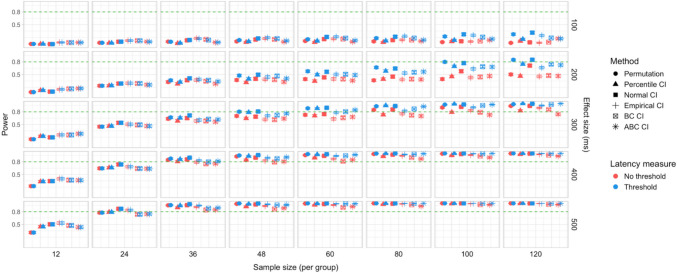
Study 2: Power estimates for analyses that calculated effect latency based on significance in consecutive time bins

**Fig. 16:**
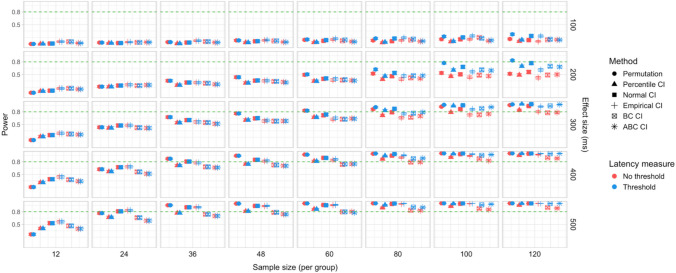
Study 2: Power estimates for analyses that calculated effect latency based on earliest effect after application of Holm–Bonferroni correction

**Fig. 17:**
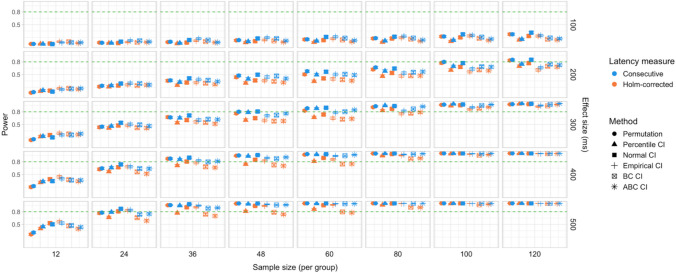
Study 2: Power comparison between latency measures based on significance in consecutive time bins and measures based on earliest effect after the application of Holm–Bonferroni correction, both with an effect size threshold

**Fig. 18:**
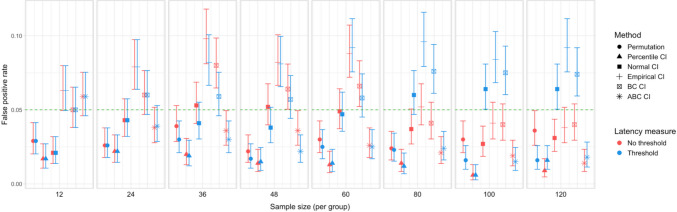
Study 2: Type I error for analyses that calculated effect latency based on significance in consecutive time bins

**Fig. 19:**
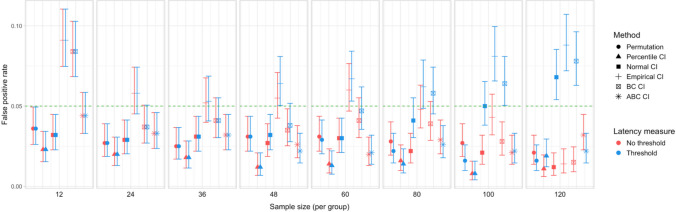
Study 2: Type I error rates for analyses that calculated effect latency based on earliest effect after the application of Holm–Bonferroni correction

**Fig. 20:**
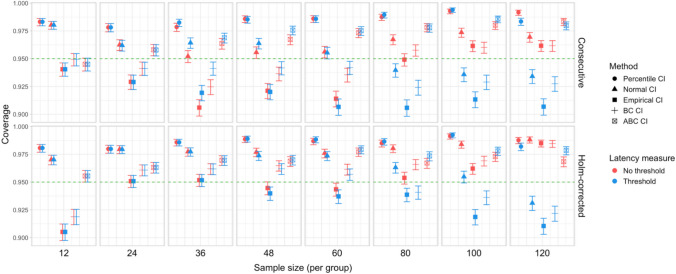
Study 2: Coverage of bootstrap confidence intervals based on data pooled across effect sizes

A total of 48,000 simulations were run (1,000 simulations for each combination of six effect sizes and eight group sample sizes), amounting to 144 million resampled datasets, and over 11.5 billion model fits. Power estimates for 960 distinct parameter combinations and Type I error rates for 192 parameter combinations were obtained. The full results are presented in Tables 3a and b and Table 4 in Appendix A, and Figs. 15, 16, 17, 18, 19, and 20.

#### Sample sizes and effect sizes

Figures 15 and 16 illustrate power estimates for varying sample sizes and effect sizes. Compared to infrared eye-tracking data analyzed in Study 1, the power to detect latency differences in webcam-based eye tracking data was substantially lower. For the smallest tested effect size of 100 ms, none of the tests approached 80% power even with group samples of 120 participants. For latency differences of 200 ms, the permutation test achieved 80% power with groups of size *n = *100 when the latency estimates were based on consecutive time with an effect size threshold (estimated power = 79.9%; 95% CI [77.3, 82.3]), and approached 80% power with the same sample size when the latency estimates were based on significance after Holm–Bonferroni correction, once again with an effect size threshold (estimated power = 77.4%; 95% CI [74.7, 79.9]). The bootstrap procedure with normal CIs and consecutive bin latency measures approached 80% power with groups of size *n = *100 when an effect size threshold was applied (estimated power = 77.1%; 95% CI [74.4, 79.6]). Several tests were able to detect latency differences of 300 ms with adequate power for samples of *n = *48 when latencies were calculated based on consecutive time bins with an effect size threshold. For most variants of the procedures, a group size of 36 participants was sufficient to achieve 80% when testing for differences of 400 ms, and 24 participants per group was sufficient to detect differences of 500 ms.

#### Latency measures

Similarly to Study 1, higher power was achieved when an effect size threshold was used in estimating latencies, especially when the true latency difference was 200 or 300 ms and the groups were relatively large (over 48 participants per group). We also observed a power advantage of using latency measures based on consecutive time bins compared to measures based on significance after family-wise error rate correction, especially in combination with bootstrapping-based tests (Fig. 17).

#### Tests

Overall, the permutation test and normal CI bootstrap tended to have higher power than the other procedures, especially for detecting small latency differences (200 ms) or when the group samples were smaller than 80 participants.

#### Type I error rates

Figures 18 and 19 illustrate the estimated Type I error rates for the tested parameter settings. As in Study 1, we found that bootstrapped empirical CIs were anti-conservative, significantly exceeding the nominal level under multiple settings. This was also the case for bootstrapped bias-corrected CIs. The bootstrap procedure with normal CIs also exceeded the nominal level in combination with the largest group sizes when an effect size threshold was applied in latency estimation. Notably, the permutation test never exceeded the nominal false-positive rate despite exhibiting higher power to detect true effects compared to most of the other tests.

#### Coverage

The overall coverage of the bootstrap CIs pooled across effect sizes is shown in Fig. 20. Percentile and ABC CIs exceeded 95% coverage for all combinations of group size and choice of latency measure. In contrast to Study 1, other types of confidence intervals tended to have lower coverage when combined with latency measures involving an effect size threshold.

### Discussion

Overall, the power of the tests to detect true differences in effect latency was substantially lower than in Study 1. Samples of at least 100 participants per group were necessary to detect a latency difference of 200 ms with 80% power, 48 participants per group to detect a difference of 300 ms, 36 participants per group to detect a difference of 400 ms, and at least 24 participants per group to detect a difference of 500 ms. Even 120 participants per group was not sufficient to reliably detect latency differences of 100 ms.

Similarly to Study 1, we found a significant increase in power in multiple configurations associated with the application of an effect size threshold in the calculation of latencies, both when latency was measured based on consecutive time bins and when it was taken as the earliest effect after family-wise error correction. For the bootstrap-based tests, measuring latency based on consecutive time bins was associated with higher power than identifying the earliest effect after correction.

Tests based on empirical, normal, and BC confidence intervals all significantly exceeded the nominal 5% level at least in some of the configurations. In contrast to Study 1, the false-positive rates of these tests tended to be higher when an effect size threshold was applied. On the other hand, the permutation test and tests based on percentile and ABC CIs maintained false-positive rates at or below the nominal level under all parameter settings. Percentile CIs also exhibited the best coverage in most configurations.

## Study 3: Infrared eye-tracking, grammatical cue (Dataset C)

In Study 3, we evaluated the latency analysis procedures when applied to a target preference effect triggered by a grammatical cue—specifically, gender on a possessive pronoun which was predictive of the following noun.

### Simulation protocol

The simulation protocol was the same as in Studies 1 and 2, with two exceptions: First, given the smaller sample size of Dataset C, we tested groups of 12, 24, and 34 participants (per group). Second, proportions of looks to the target picture were calculated in fifty 40-ms time bins following the onset of the possessive pronoun, rather than forty 50-ms time bins as in Studies 1 and 2. This bin size was selected because the data in Dataset C had been downsampled to 50 Hz, i.e., one data point every 20 ms. With 40-ms bins, data points could be evenly distributed between time bins (two data points per bin) while keeping the bin size close to that used in Studies 1 and 2.

### Results

**Fig. 21:**
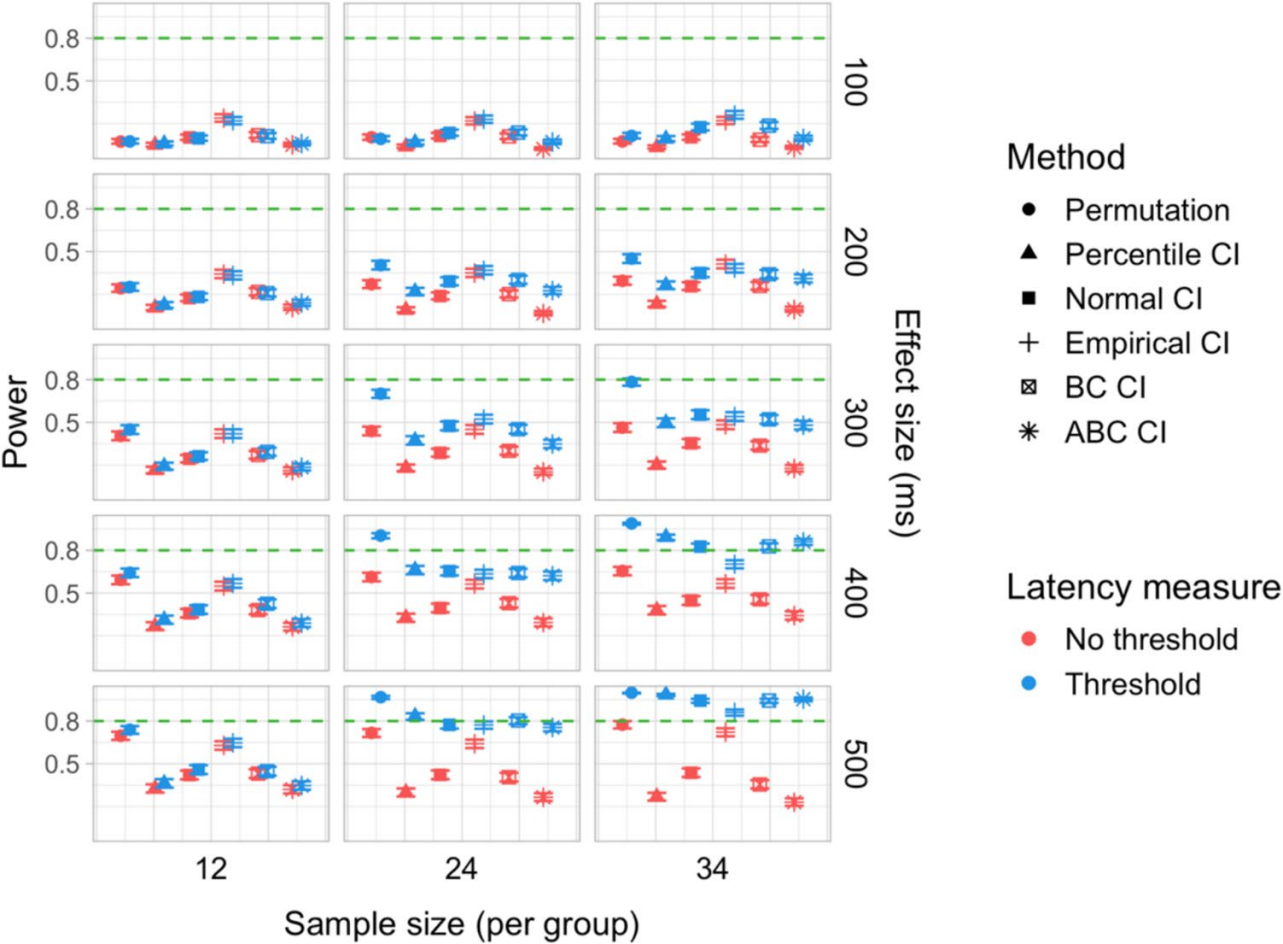
Study 3: Power estimates for analyses that calculated effect latency based on significance in consecutive time bins

**Fig. 22:**
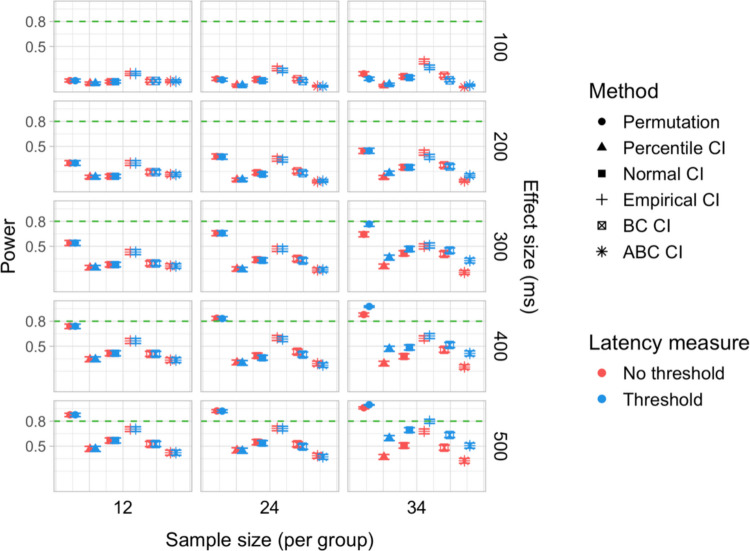
Study 3: Power estimates for analyses that calculated effect latency based on earliest effect after application of Holm–Bonferroni correction

**Fig. 23:**
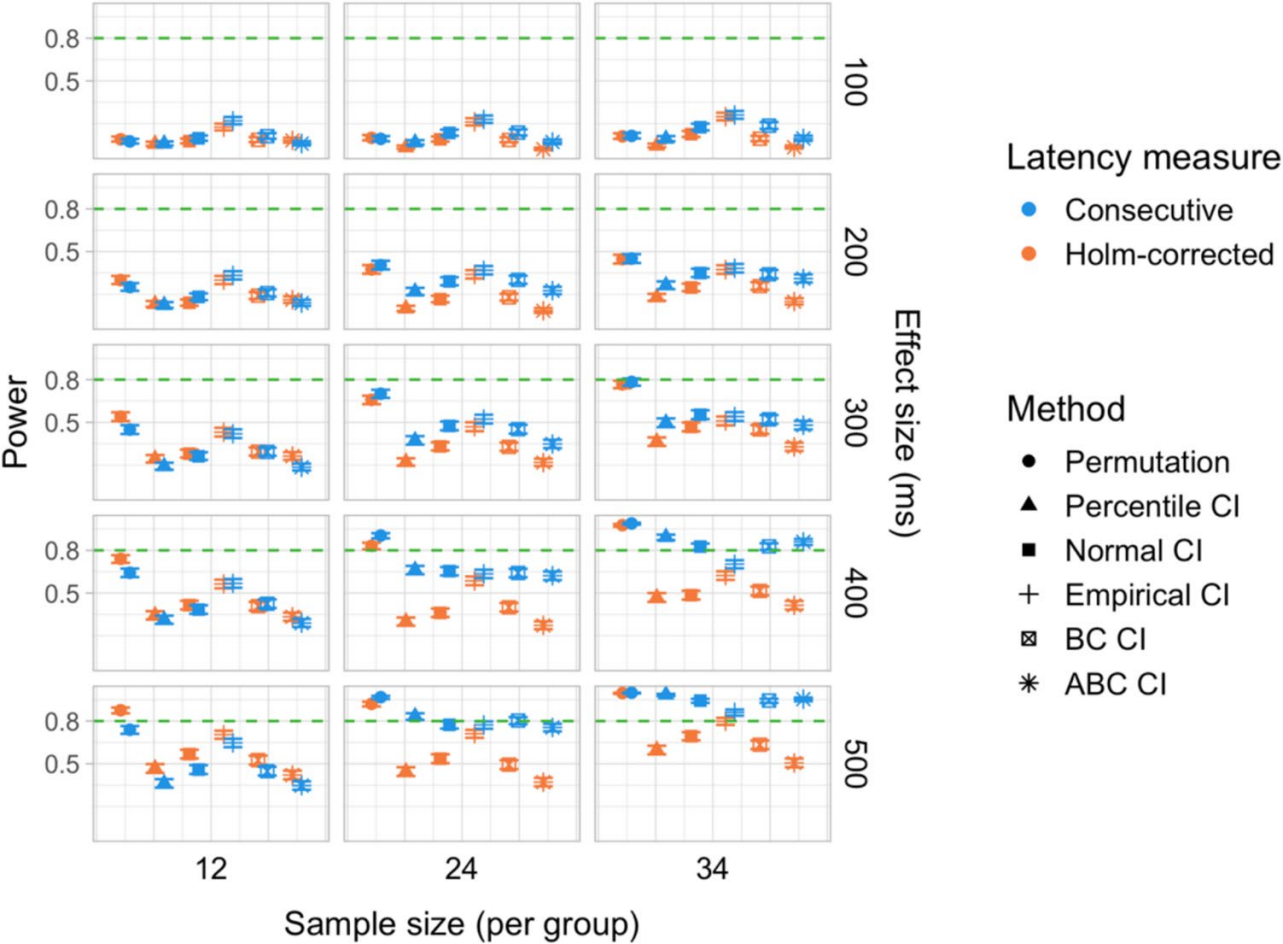
Study 3: Power comparison between latency measures based on significance in consecutive time bins and measures based on earliest effect after application of Holm–Bonferroni correction, both with an effect size threshold

**Fig. 24:**
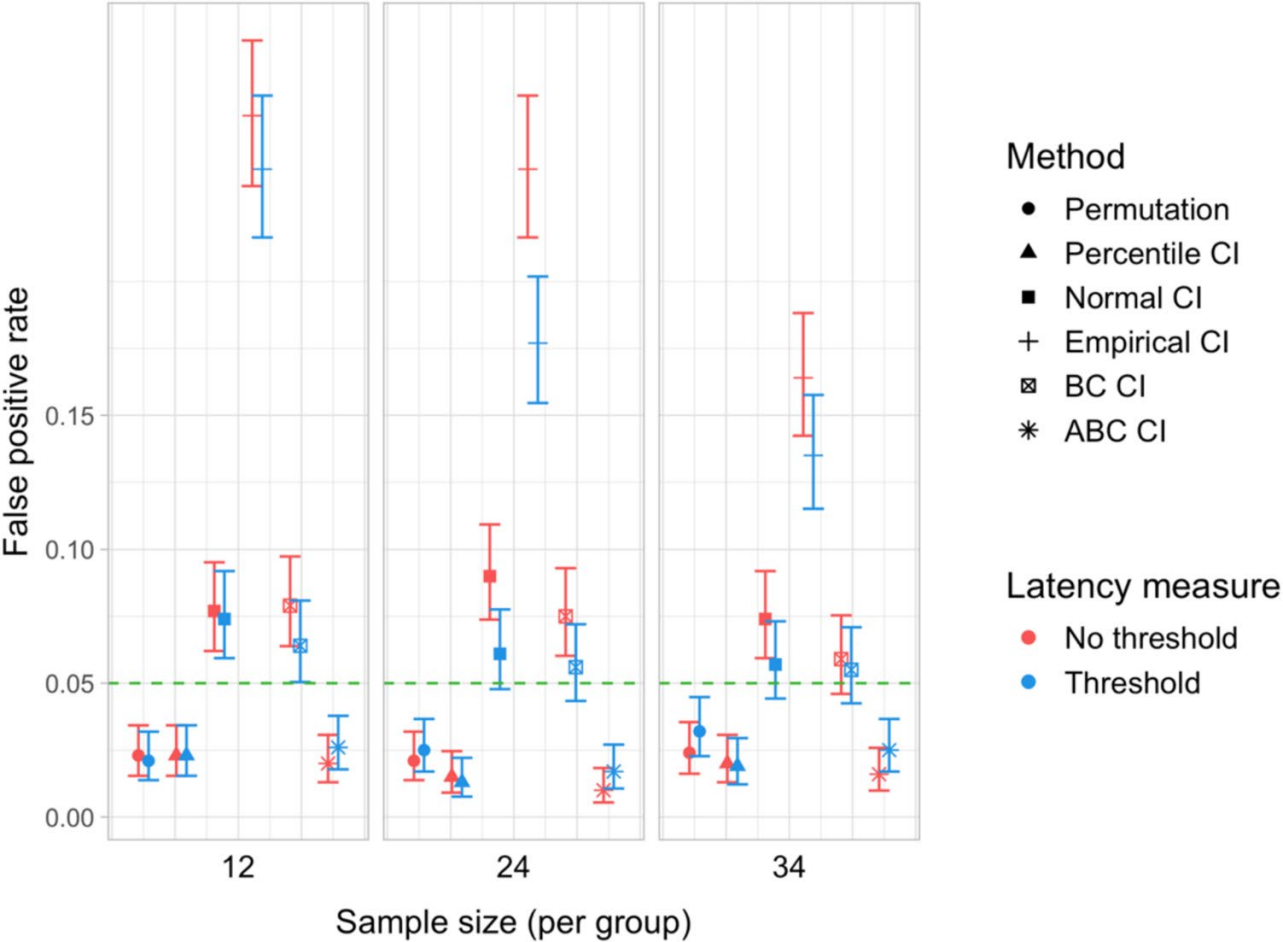
Study 3: Type I error rates for analyses that calculated effect latency based on significance in consecutive time bins

**Fig. 25:**
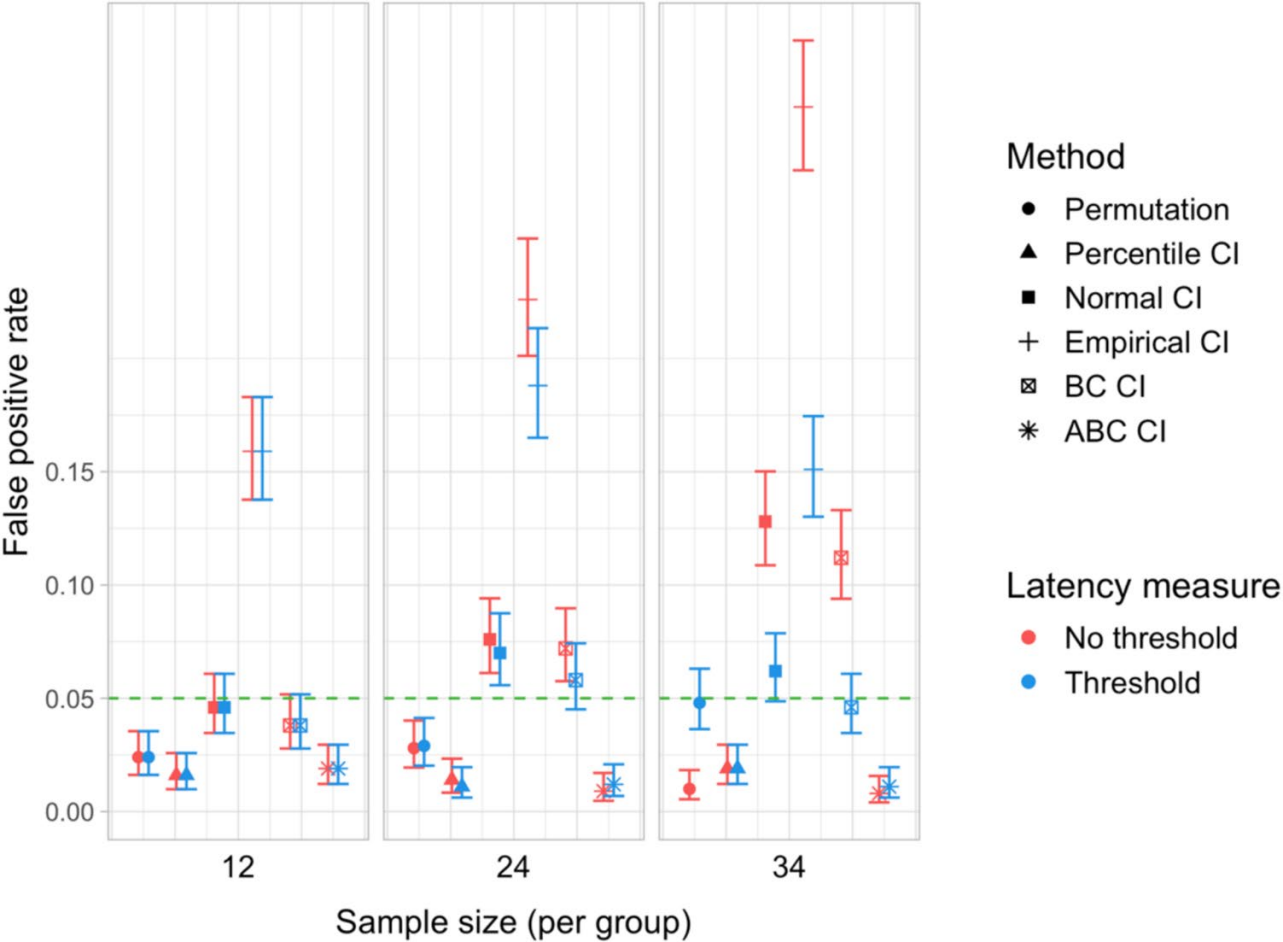
Study 3: Type I error rates for analyses that calculated effect latency based on earliest effect after application of Holm–Bonferroni correction

**Fig. 26:**
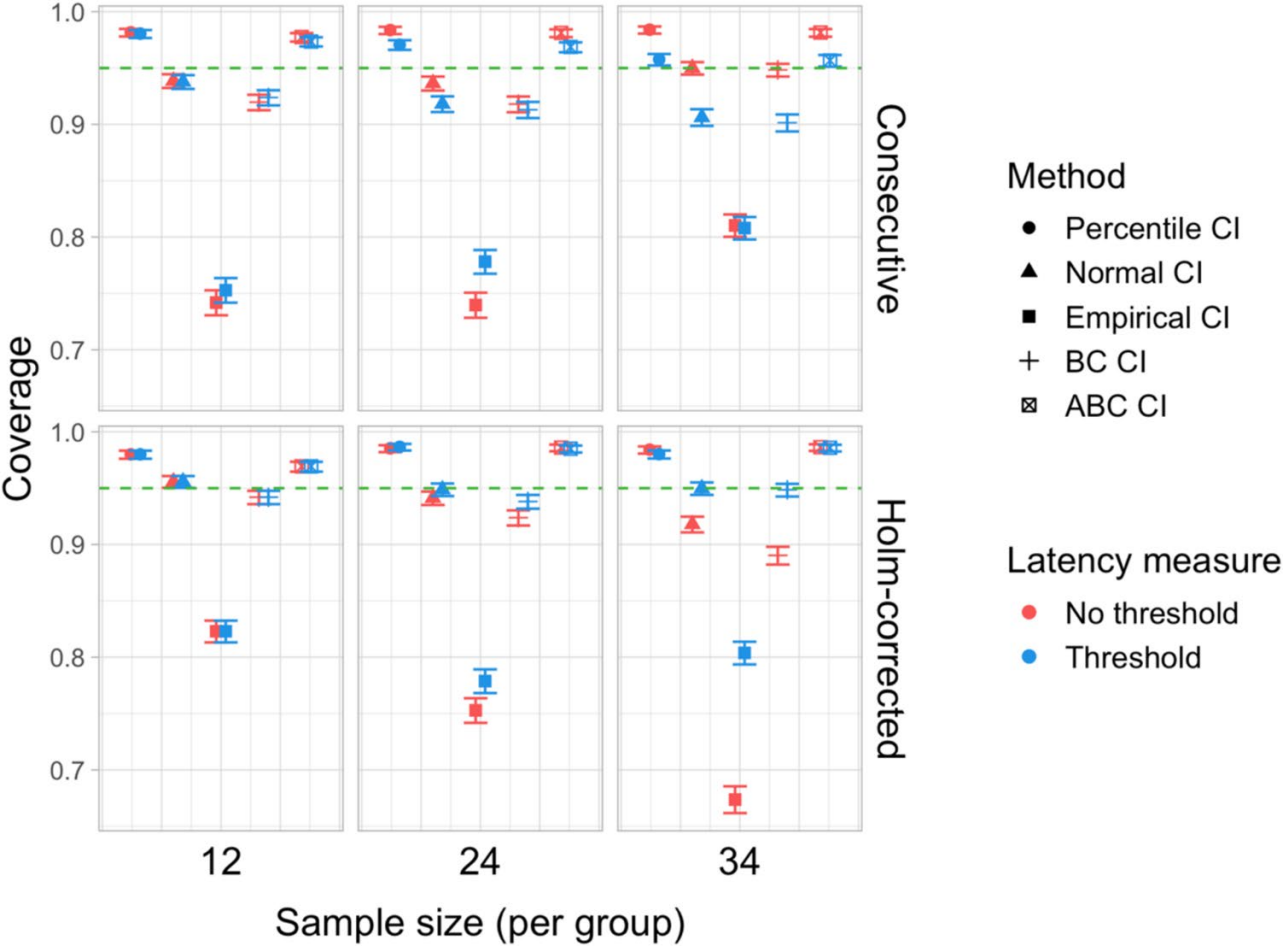
Study 3: Coverage of bootstrap confidence intervals based on data pooled across effect sizes

We conducted 18,000 simulations, which involved 54 million resamples and 5.4 billion model fits. We obtained power estimates for 360 parameter combinations and Type I error rates for 72 parameter combinations. The full results are presented in Tables 5 and 6 in Appendix A and in Figs. 21, 22, 23, 24, 25, and 26.

#### Sample sizes and effect sizes

As expected, the power to detect differences in effect latencies varied depending on participant sample size and true effect size; see Figs. 21 and 22. None of the tests approached 80% in detecting latency differences of 100 and 200 ms. However, already for 300-ms effects, permutation tests approached 80% power for group samples of 24 participants, and achieved 80% power for samples of 34 participants, when applied to latency measures employing an effect size threshold and/or based on significance after Holm–Bonferroni correction. For 400 ms latency differences, the same permutation tests achieved 80% power for sample sizes of *n = *24, while multiple bootstrap-based tests achieved 80% power for *n = *34, when applied to latency measures based on consecutive bins with an effect size threshold. When the true effect size was 500 ms, the same tests achieved 80% power for *n = *24.

#### Latency measures

We observed a substantial increase in power when an effect size threshold was used for effect latency estimation. This advantage was more pronounced for larger group sizes and for latency measures based on consecutive time bins. However, when comparing between latency measures based on consecutive time bins and those based on significance after Holm–Bonferroni correction, we also found a noticeable advantage in using the former, especially for the bootstrap-based tests (Fig. 23). In other words, overall, latency measures based on significance in consecutive time bins with an effect size threshold afforded the highest power to detect differences in effect latency.

#### Tests

Similarly to Study 2, but to an even greater extent, we found that permutation tests exhibited higher power to detect latency differences than the bootstrap-based procedures.

#### Type I error rates

We found that bootstrapped empirical CIs were consistently anti-conservative for all combinations of group and effect sizes, with the false-positive rate in certain cases exceeding 25%. Tests based on normal and bias-corrected bootstrapped CIs also significantly exceeded the nominal level under certain conditions. In contrast, permutation tests and bootstrap-based tests using percentile and accelerated bias-corrected CIs never exceeded the nominal level (Figs. 24 and 25).

#### Coverage

The overall coverage of the bootstrap CIs is illustrated in Fig. 26. Consistent with the results of Studies 1 and 2, percentile CIs remained above 95% coverage across all combinations of group size and latency measure. ABC CIs performed similarly well. Other bootstrap CIs had lower coverage, with empirical CIs performing the worst.

### Discussion

The permutation tests exhibited the highest power: group size of 34 participants per group was sufficient to reliably detect differences in latency of 300 ms, and 24 participants per group for effects of 400 and 500 ms. Tests based on bootstrap confidence intervals required groups of 34 participants to detect differences of 400 ms with 80% power, and groups of 24 participants to detect differences of 500 ms. None of the tests were able to detect latency differences of 100 or 200 ms with 80% power. In terms of choice of latency measure, the results were similar to Studies 1 and 2. Higher power was achieved when an effect size threshold was applied in latency calculation. For bootstrap-based tests, calculating latency based on an effect in consecutive times resulted in higher power than measuring it based on the earliest effect after family-wise error correction.

With respect to Type I error rates, the permutation test and the percentile and ABC bootstrap CIs stayed at or under the nominal 5% in all the tested configurations. When an effect size threshold was applied in latency calculation, BC CIs also did not significantly exceed the 5% rate. In contrast, normal and, especially, empirical CIs exceeded the nominal level in multiple tested configurations, with and without the application of an effect size threshold. Percentile and ABC CIs showed the highest coverage.

## Study 4: Cohort effects in word recognition (Dataset D)

In Study 4, we tested the latency analysis procedures when applied to cohort effects during word recognition based on the data from Apfelbaum et al. (2021) (Dataset D).

### Simulation protocol

The simulation protocol was the same as in Studies 1–3, with the following modifications:

#### Resampling procedure and group sizes

To control for variation associated with the five different preview conditions used in Apfelbaum et al.’s (2021) experiment, in each simulation we sampled an equal number of participants from each condition in each of the compared groups (Baseline and Shifted). This also meant that the group sizes needed to be divisible by 5, so we tested groups of 15, 25, 35, 45, and 55 participants (per group). Similarly, to control for the differences in preview type, permutation and bootstrap resampling were performed *within* the preview conditions, thus preserving an equal number of participants from each condition in each group.

#### Effect sizes

Word recognition tasks are typically more controlled than sentence processing in several respects: the participants’ gaze patterns are not influenced by the processing of linguistic context preceding the critical word, gaze direction prior to the onset of the critical word is usually controlled (e.g., by displaying a cross in the middle of the screen prior to word presentation), and participants have strong expectations of hearing the name of one of the items in the display which may be previewed. We may thus expect smaller latency differences to be relevant in word recognition research, and moreover, we expected the latency comparison procedures to be more powerful in detecting latency differences due to reduced variation. Based on these considerations, in Study 4 we tested smaller latency differences of 50, 100, and 200 ms (as well as 0 to obtain Type I error rates).

#### Latency measures

The publicly shared dataset from Apfelbaum et al. (2021) contains proportions of looks to the target, cohort competitor, and unrelated pictures averaged by participant and time point, with time points sampled every 4 ms (the proportions of looks to unrelated pictures were obtained by averaging the looks to the two unrelated objects in the display). In the simulations, we further downsampled the data to 40-ms time bins so that the results would be comparable to Studies 1–3. However, since the dataset did not contain data from individual trials, we could not use logistic regression models to identify time bins with significant effects. Instead, to identify time bins where there were more looks to the cohort competitor than the unrelated pictures (our effect of interest), we calculated the relative proportion of looks to the competitor versus unrelated pictures for each participant and time bin, taken as competitor proportion/(competitor proportion + unrelated proportion). Then, for each time bin, we applied a *t-*test to see whether the relative proportion of looks to the cohort competitor was significantly above 50%. The same four latency measures as in Studies 1–3 were then obtained:

- Earliest of five consecutive time bins with a significant effect
- Same as previous but with an effect size threshold, which was set as a > 0.03 difference between the proportion of looks to the cohort competitor and the proportion of looks to unrelated pictures (this corresponds to approximately 57% relative proportion of looks to the cohort competitor, close to the 55% threshold used in Studies 1–3)
- Earliest time bin with a significant effect after application of the Holm–Bonferroni correction
- Same as previous but combined with an effect size threshold

### Results

**Fig. 27:**
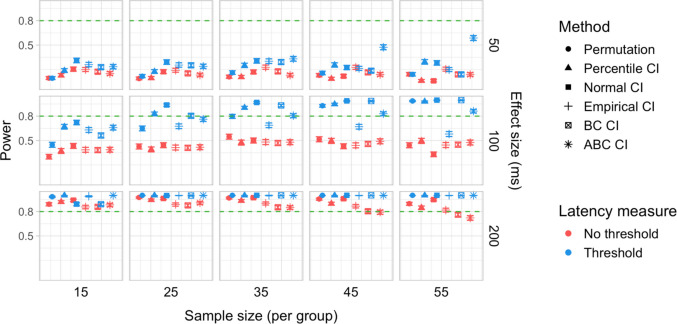
Study 4: Power estimates for analyses that calculated effect latency based on significance in consecutive time bins

**Fig. 28:**
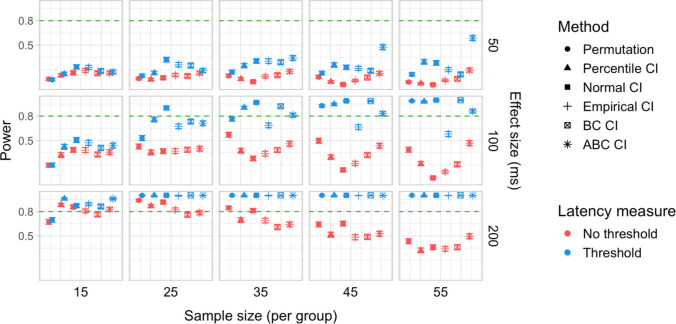
Study 4: Power estimates for analyses that calculated effect latency based on earliest effect after application of Holm–Bonferroni correction

**Fig. 29:**
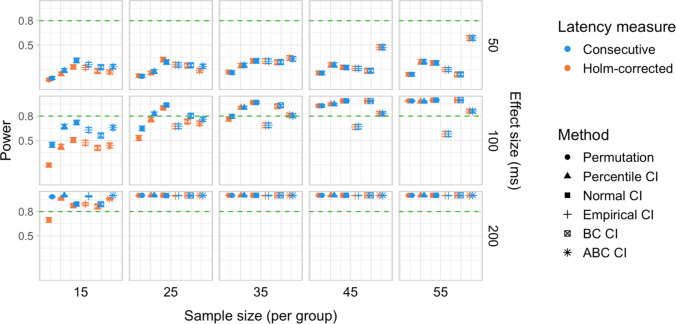
Study 4: Power comparison between latency measures based on significance in consecutive time bins and measures based on earliest effect after application of Holm–Bonferroni correction, both with an effect size threshold

**Fig. 30:**
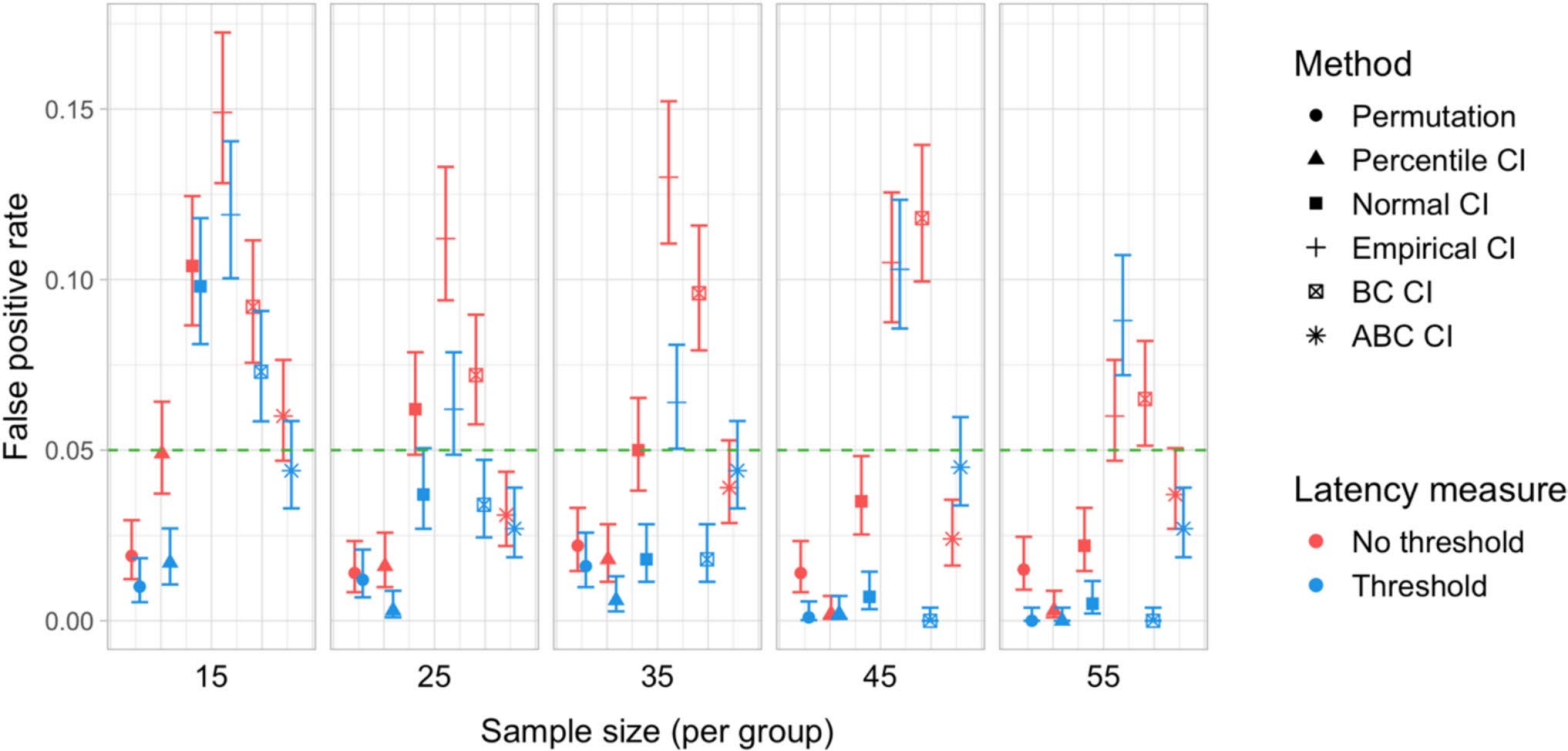
Study 4: Type I error rates for analyses that calculated effect latency based on significance in consecutive time bins

**Fig. 31:**
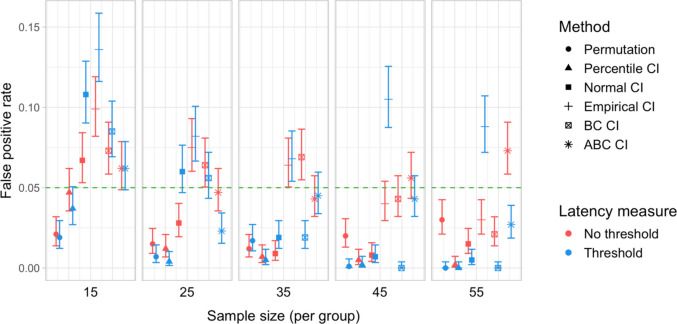
Study 4: Type I error rates for analyses that calculated effect latency based on earliest effect after application of Holm–Bonferroni correction

**Fig. 32:**
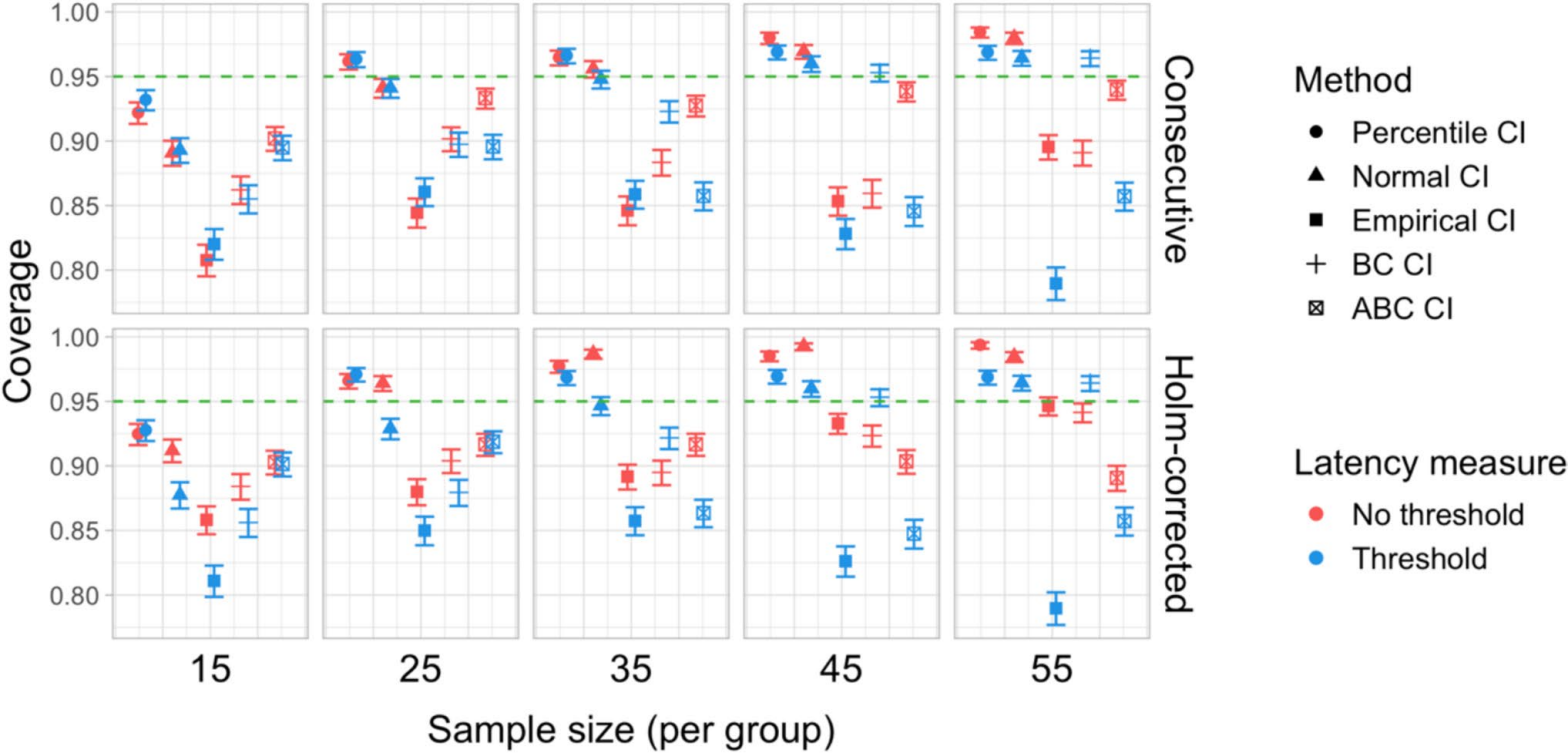
Study 4: Coverage of bootstrap confidence intervals based on data pooled across effect sizes

A total of 20,000 simulations were conducted, which involved 60 million resamples and 4.8 billion statistical tests. We obtained power estimates for 360 parameter combinations and Type I error rates for 120 parameter combinations. The full results are presented in Tables 7 and 8 in Appendix A and in Figs. 27, 28, 29, 30, 31, and 32.

#### Sample sizes and effect sizes

Overall, the power to detect small latency differences was higher for the tested cohort effects in a word recognition experiment than in Studies 1–3 in the context of sentence processing (Figs. 27 and 28). None of the tested procedures was able to detect 50-ms latency differences with 80% power even with the largest sample sizes. However, effects of 100 ms were already reliably detected under multiple parameter combinations where the latency measure involved an effect size threshold. Here, multiple bootstrap-based measures exhibited close to or above 80% power with group samples of 25 participants, while the permutation-based test required samples of at least 35 to achieve 80% power. Effects of 200 ms were reliably detected under almost all the tested configurations where the latency measures were based on effects in consecutive times bins and/or involved an effect size threshold.

#### Latency measures

As in previous studies, we found a substantial advantage in applying an effect size threshold in latency calculation (see Figs. 27 and 28). When an effect size threshold was applied, latency estimates based on consecutive time bins and those based on Holm–Bonferroni-corrected significance performed similarly under most conditions, although the former measure had an advantage with small sample sizes (Fig. 29).

#### Tests

Focusing on latency measures with an effect size threshold, bootstrap-based tests tended to have higher power than permutation-based tests, especially with smaller sample sizes. Within the bootstrap-based tests, tests based on empirical CIs tended to have lower power in multiple configurations, while tests based on ABC CIs noticeably outperformed other tests in detecting 50-ms effects with larger participant samples (although even these tests did not achieve 80% power).

#### Type I error rates

Tests based on normal, bias-corrected and, especially, empirical bootstrapped CIs were anti-conservative, significantly exceeding the nominal false-positive rate under multiple parameter combinations (Figs. 30 and 31). Tests based on accelerated bias-corrected CIs did not significantly exceed the nominal rate when used with latency measures employing an effect size threshold. Finally, the permutation-based test and test based on percentile bootstrapped CIs did not exceed the nominal rate under any of the tested parameter combinations.

#### Coverage

Figure 32 illustrates the overall coverage of bootstrapped CIs pooled across the tested effect sizes. Consistent with Studies 1–3, percentile CIs displayed the highest coverage, falling below 95% only for the smallest sample size (*n = *15), and empirical CIs displayed the lowest overall coverage. In contrast to previous results, ABC CIs had relatively low coverage across multiple configurations.

### Discussion

Compared with Studies 1–3, the power to detect small (100 ms) effects was higher. Bootstrap-based tests combined with a latency measure based on consecutive time bins with an effect size threshold exhibited the highest power, reliably detecting 100-ms effects with groups of 25 participants and 200-ms effects with groups of 15 participants. Permutation tests when combined with the same latency measure required 35 participants per group to achieve 80% power in detecting 100-ms effects, and 15 participants per group in detecting 200-ms effects. Overall, the results once again indicated a substantial power advantage in applying an effect size threshold in latency calculation. With respect to Type I error rates, consistent with previous results, percentile bootstrapped CIs and permutation-based tests were the most reliable, never exceeding the nominal false-positive rate.

## Study 5: False-positive rates under increased variability and comparison of sampling methods

Study 5 pursued two goals. The first was to test the robustness of the latency analysis procedures to increased cross-participant variation in effect timing. Specifically, we wanted to test whether the false-positive rate of the latency comparison tests would stay below the nominal level if inter-speaker variation in effect timing was increased. Our second aim was to compare the by-participant bootstrapping procedure adopted in Studies 1–4 to the stratified bootstrapping procedure from Stone et al. (2020), which involves resampling within participants and time bins (see also Stone et al., 2021; Ito & Knoeferle, 2023). As noted in the introduction, the stratified resampling procedure may not fully control for cross-speaker variability, since each resampled dataset represents data from the same array of participants. Consequently, we expected that it could lead to excessive false-positive rates in the latency comparison tests, and moreover that these rates might rise further with increased cross-speaker variability.

### Simulation protocol

Simulations were based on subsamples from Dataset A and Dataset C. For Dataset A, we ran simulations for each combination of group size *n* of 12, 24, 36, 48, and 60 participants and *added noise level* of 0, 50, 100, and 200 ms, corresponding to the standard deviation of white noise added to the participants’ time stamps (see below). For Dataset C, we conducted simulations for group sizes of 12, 24, and 34 participants, and the same range of added noise values. Furthermore, at the request of a reviewer, we also tested larger group sizes of 48 and 60 participants per group. In this case, we used resampling *with replacement* to obtain the simulated datasets, since the total number of participants in Dataset C (*n = *69) was not sufficient to obtain large enough group samples without replacement.

Since we were interested in the false-positive rate, the true effect size was kept at 0. For each parameter combination, we conducted 1,000 simulations. Each simulation followed the same sequence of steps as Study 1, with two exceptions:

In Step 1, we randomly sampled two groups of *n* participants and divided them into the Baseline group and the Shifted group, as in Study 1. Then, for each participant, we sampled a random noise value from a normal distribution with mean 0 and standard deviation equal to 0, 50, 100, or 200 ms, and added this value to all participants’ time indices. This increased the inter-participant variation in response latency within both groups, while retaining the true latency difference between the groups at 0.

Step 5 (additional step): In addition to conducting a by-participant bootstrap on each simulated dataset (as in Studies 1–4), we ran a *stratified bootstrap*. Once again, for each participant sample, a random noise value (*SD* equal to 0, 50, 100, or 200 ms) was added to the participants’ time indices, and we used the *boot* function from the *boot* package in R to reproduce the sampling procedure from Stone et al. (2020). This involved stratifying the simulated dataset by group, participant, and time bin, and resampling 2,000 times with replacement within these strata. For each bootstrap sample, we obtained estimates of the difference in effect latency between the groups. We then used the bootstrap distributions of these differences to obtain 95% CIs, applying the same range of methods as for the by-participant bootstrap.

### Results

**Fig. 33:**
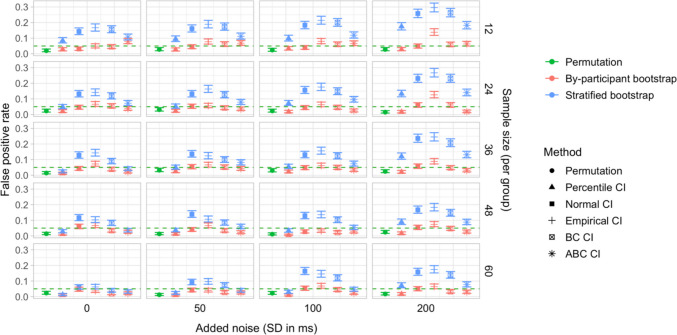
Study 5a: Type I error rates for comparisons of latency measures based on significance in consecutive time bins with an effect size threshold (data sampled from Dataset A)

**Fig. 34:**
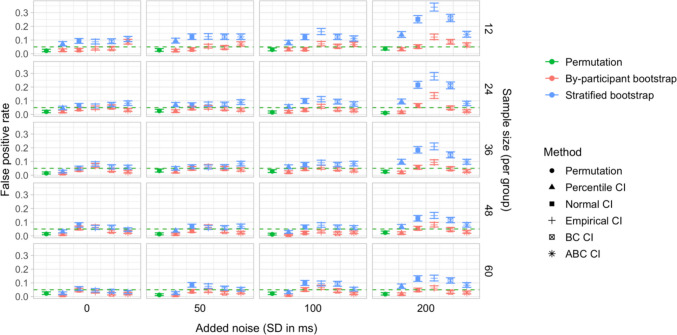
Study 5a: Type I error rates for comparisons of latency measures based on earliest significance after Holm–Bonferroni correction with an effect size threshold (data sampled from Dataset A)

**Fig. 35:**
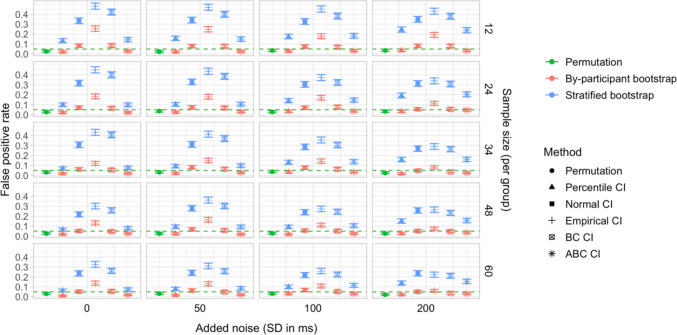
Study 5b: Type I error rates for comparisons of latency measures based on significance in consecutive time bins with an effect size threshold (data sampled from Dataset C)

**Fig. 36:**
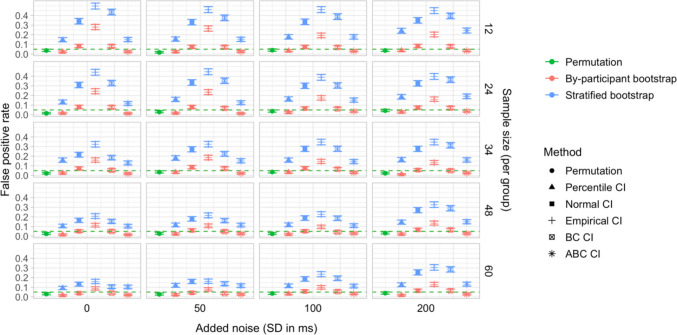
Study 5b: Type I error rates for comparisons of latency measures based on significance in consecutive time bins without an effect size threshold (data sampled from Dataset C)

A total of 40,000 simulations were conducted: 1,000 simulations for each of 20 parameter combinations with data subsampled from Dataset A and Dataset C. Each simulation involved 1,000 permutations, 2,000 by-participant bootstrap resamples, and 2,000 stratified bootstrap resamples, amounting to 200 million resampled datasets and 18 billion model fits. The full results are presented in Tables 9a and b and 10a and b in Appendix A. Figures 33 and 34 illustrate the obtained false-positive rates for simulations based on Dataset A for latency measures with an effect size threshold. The results for procedures without a threshold were very similar. Figures 35 and 36 illustrate the results of simulations based on Dataset C, with and without an effect size threshold.

#### Permutation and by-participant bootstrap

The permutation test proved robust against increases in inter-participant variability, never exceeding the nominal level in any of the tested configurations (Figs. 33, 34, 35, and 36). Tests based on the by-participant bootstrap exhibited varying degrees of conservativeness. Empirical bootstrap CIs were the least conservative. Here, the false-positive rates exceeded the nominal level under maximal inter-speaker variability in simulations based on Dataset A, and under most parameter combinations in simulations based on Dataset C. Normal, bias-corrected, and accelerated bias-corrected CIs also exceeded the nominal false-positive rate under some of the tested conditions. In contrast, percentile CIs never exceeded the nominal false-positive rate under any of the tested conditions.

#### Stratified bootstrap

As expected, tests based on the stratified bootstrap exhibited higher false-positive rates than tests based on the by-participant bootstrap. For simulations based on Dataset A, all tests exceeded the nominal false-positive rate at least for some of the parameter combinations. The percentile CI test was the most conservative, but it also significantly exceeded the 5% false-positive rate in multiple configurations involving smaller group sizes and/or higher levels of inter-speaker variability (Figs. 33 and 34). For simulations sampled from Dataset C, the percentile CI test was once again the most conservative, but even this test significantly exceeded the nominal false-positive rate in almost all the tested configurations (Figs. 35 and 36).

### Discussion

Overall, the permutation test and by-participant bootstrap percentile CIs proved robust against increased inter-speaker variation, never exceeding the nominal false-positive rate under any of the tested parameter combinations. In contrast, all tests based on the stratified bootstrap sampling within participants and time bins were found to be anti-conservative.

## General discussion

In a series of studies, we assessed the power and Type I error rates of bootstrapping- and permutation-based procedures for comparing effect latencies between groups in the VWP. We conducted Monte Carlo simulations to assess the performance of these procedures under various conditions. We manipulated several characteristics of the simulated datasets—such as the type of effect, data collection method, sample size, true effect size, and degree of between-subject variability—as well as key procedural parameters, including the choice of latency measure, statistical test, and resampling method. Across the five studies, a total of 156,000 simulations were conducted, involving 548 million resampled datasets and 46.9 billion model fits.

**Fig. 37:**
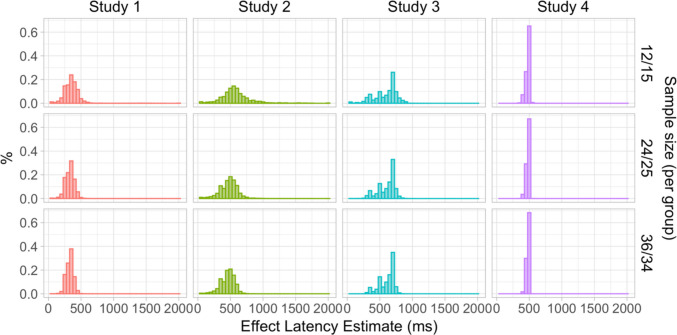
Histograms of effect latency estimates based on pooled bootstrap resamples for the Baseline group across all tested effect sizes. Latencies were calculated based on a significant effect in consecutive time bins with an effect size threshold

Our results suggest that resampling-based methods for between-group latency comparison in VW experiments can detect differences as small as 200 ms in sentence processing tasks, and as small as 100 ms in word recognition tasks, with above 80% power—given sufficient sample sizes and under specific choices of tests and latency measures, and subject to variation depending on the properties of the analyzed data. We found that highest power was achieved for data sampled from Dataset D (Study 4), constituting the results of a word recognition experiment. In the context of sentence processing tasks, highest power was achieved for data sampled from Dataset A (Study 1). Power was substantially lower (controlling for group and effect sizes) for data sampled from Datasets B and C (Studies 2 and 3). Correspondingly, we found that the variability of latency estimates was lowest for Dataset D, and was lower for Dataset A than for Datasets B and C. Figure 37 illustrates the sampling distributions of latency estimates in Studies 1–4 for group sizes of 12/15, 24/25, and 34/36 participants. Latencies were calculated based on effect in consecutive time bins with an effect size threshold, and were taken from all bootstrap resamples for the Baseline group pooled across the tested effect sizes—amounting to 12 million latency values for each study and group size. Variation in effect latencies was lowest in Study 4, which tested cohort competitor effects in a word recognition task ($\widehat{SE}=\hspace{0.17em}33.9$ for group size 15, $\widehat{SE=27.4}$ for group size 25, $\widehat{SE}= 23.8$ for group size 35). Among the studies that tested target preference effects in sentence processing, Study 1 had the lowest variation in effect latencies ($\widehat{SE }= 143.6$ for group size 12, $\widehat{SE = 72.4}$ for group size 24, $\widehat{SE }= 56.2$ for group size 36). Variation was higher in Study 2 ($\widehat{SE = 242.9}$ for group size 12, $\widehat{SE}=\hspace{0.17em}137.7$ for group size 24, $\widehat{SE }= 109.1$ for group size 36), and also in Study 3—despite a larger item sample ($\widehat{SE}\hspace{0.17em}=$171.2 for group size 12, $\widehat{SE }= 128.8$ for group size 24, $\widehat{SE}=\hspace{0.17em}111.74$ for group size 34).

Datasets A and B shared the same study design but differed in the method of data collection. While the experiment for Dataset A was conducted in person using an infrared eye tracker, Dataset B was collected using webcam-based eye tracking over the web. The observed increased variability in effect latencies in Dataset B is likely to due to a combination of technological and environmental factors (Semmelmann & Weigelt, 2018). Webcam images provide less precise information than images acquired by infrared eye trackers, which use infrared light reflected from the cornea to precisely determine the point of gaze. This inevitably adds more noise to the webcam eye-tracking data. Moreover, in a web-based study, participants use their own computers, which increases the variability in the quality of the webcam images, the sampling rate, and the performance of the algorithms that are run to calculate the gaze direction. Finally, in a web-based experiment, different participants perform the task in different settings (with differing levels of lighting, distractions, etc.), which may also contribute to increased variance in the results. Thus, Slim and Hartsuiker (2023) conducted an online webcam-based VW eye-tracking study and observed considerable variability between participants in the timing of looks to the target picture. Similarly, Semmelmann and Weigelt (2018) found that webcam eye-tracking data collected online showed higher variance than webcam data collected in a lab setting. Our results are in line with these previous findings.

The difference between Datasets A and C, on the other hand, is likely due to the nature of the studied effect. Dataset A was based on filler items where target preference was triggered by the processing of (the onset of) a lexical item—the verb, which matched one of the presented pictures. Target preference in Dataset C, on the other hand, reflects an experimental effect where participants displayed predictive looks to the referent of an upcoming noun based on the gender inflection of a possessive pronoun (i.e., the target preference effect preceded the presentation of the noun). Figure 37 suggests both multimodality and increased variability in the distribution of effect latencies in Dataset C, which may indicate the presence of distinct patterns of predictive processing among the speakers. The nature and source of variation in predictive processing warrant further research.

Finally, the relatively low variation in effect latencies in Dataset D is likely due to the more controlled nature of the word recognition experiment compared to sentence processing tasks. Unlike sentence processing, in a word recognition task, participants’ gaze patterns at the critical item are not influenced by preceding context. Instead, gaze direction prior to the onset of the critical word was controlled by displaying a fixation cross in the center of the screen, and participants always knew that the upcoming word would uniquely identify one of the pictures. Furthermore, the experiment involved a much larger number of trials per participant (192) compared to typical sentence processing experiments (24 in Studies 1 and 2; 48 in Study 3). All these factors likely contributed to the low variation in effect latencies observed and, consequently, to the higher power of the tested procedures to detect latency differences.

In Studies 1–4, we compared the power and false-positive rates of several resampling-based procedures for testing differences in effect latencies between groups in the VWP. In terms of Type I error rates, tests based on permutation and percentile bootstrap CIs proved the most robust, consistently staying within the nominal 5% rate across all tested configurations. Accelerated bias-corrected CIs performed well overall, only occasionally exceeding the nominal Type I error rate with the smallest group sizes (cf. the results of Study 1 and Study 3). Tests based on normal and bias-corrected bootstrap CIs were anti-conservative under multiple parameter combinations. Finally, empirical bootstrap CIs were the least conservative, with the false-positive rate consistently exceeding 5%, and going as high as 25% under certain conditions. Given these results, in the following we focus on the permutation test and percentile bootstrap CIs.

**Fig. 38:**
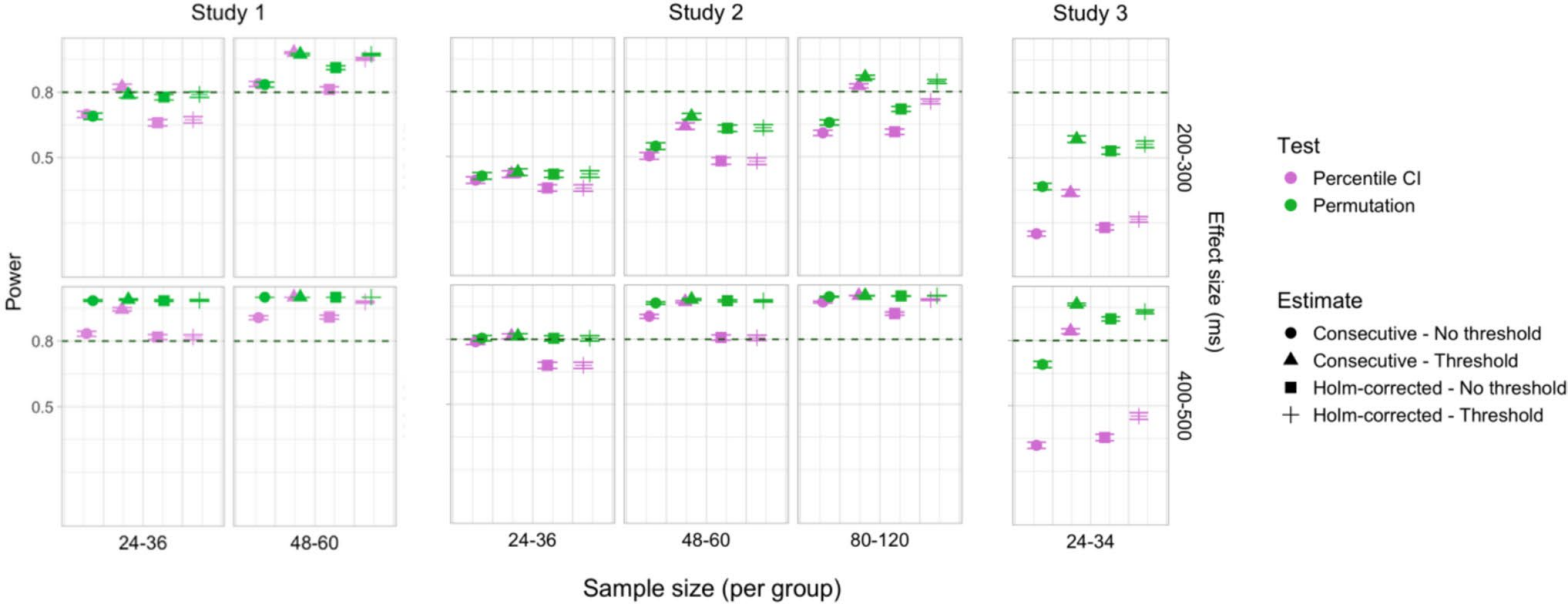
Studies 1–3: Averaged power estimates for the permutation test and bootstrap percentile confidence intervals by group and effect size (smallest group and effect sizes excluded)

In Study 1, the permutation test and percentile bootstrap CIs demonstrated comparable power to detect true differences in latency.^6^ In Study 2, the permutation test had an advantage when latencies were measured as the earliest effect after Holm–Bonferroni family-wise error correction. In Study 3, the permutation test markedly outperformed percentile CIs under many combinations of group and effect sizes and choice of latency measure. These results are summarized in Fig. 38.

These results are consistent with the general observation that permutation tests tend to be more powerful than the non-parametric bootstrap (Good, 1994, p. 20). However, it is important to keep in mind that the validity of permutation tests relies on the assumption of *exchangeability under the null hypothesis*, whereby the joint distribution of the observations is invariant under label permutations if the null hypothesis is true (Good, 1994, 2005). In our case, this means that if there is no difference in effect latency between the groups, randomly exchanging group labels should not affect the probability of the data. This assumption may be satisfied or not depending on the properties of the compared groups and the study design. For instance, if there is reason to believe that certain aspects of the data (e.g., the *size* of the effect of interest in the gaze patterns) may differ between the populations even if the latency of the effect is the same, the exchangeability assumption may be violated (cf. Fig. 1B). Researchers should therefore carefully consider the implications of the exchangeability assumption for their data when applying the permutation test.

Study 4 produced somewhat contrasting results, with percentile bootstrap CIs outperforming the permutation test under multiple conditions involving smaller effect and group sizes (Figs. 27 and 28). With sufficient sample sizes (e.g., 45 or more participants per group), the two tests performed comparably.

With regard to the choice of latency measure, we found that the application of a minimal effect size threshold often led to an increase in test power. For percentile bootstrap CIs, the latency measure based on consecutive time bins with an effect size threshold offered significant advantages over other latency measures across the four studies and under most conditions (see Fig. 27, 28, and 38). For the permutation test, this measure also generally performed best.

While application of an effect size threshold in latency estimation appears to provide a consistent statistical advantage, it introduces an additional parameter to the analysis procedure, thus increasing the researcher degrees of freedom (Simmons et al., 2011). Consequently, in order to preserve the validity of the analysis, it is essential that the minimally relevant effect size is determined in advance, before any data analysis is conducted, and ideally given a theoretical justification.

Finally, we tested the robustness of the by-participant bootstrap procedure to increases in cross-subject variability in effect timing, and compared it to a stratified bootstrap procedure based on resampling within participants and time bins, as proposed by Stone et al. (2020). In the by-participant bootstrap, resamples are constructed by drawing *n* participants from each group with replacement, and including all the data from these participants. We found that when this resampling method was used, the false-positive rate of tests based on percentile bootstrap CIs did not exceed the nominal level even under the highest levels of cross-speaker variability. Stratified bootstrapping, on the other hand, was often associated with elevated Type I error rates, significantly exceeding the 5% level. This is likely due to the fact that resampling *within participants* means that every sample contains data from the same array of subjects (although the actual data points included in the sample may be different), and consequently, the variability in latency estimates due to inter-speaker variation is underestimated in the bootstrap distribution.

Inevitably, computation costs limited the scope of the current study in several respects. First, we focused on the application of resampling methods for comparing effect latencies between groups, and did not investigate latency comparison between conditions in a within-subject design. Overall, we might expect that the power of the tests would be higher in within-subjects designs due to the reduced effect of cross-subject variability. The results of the current study may inform the choice of test in within-subject experiments: tests that have been shown to be anti-conservative when applied to between-group comparisons are to be disfavored. With regard to the choice of latency measure, we anticipate that employing an effect size threshold would provide a power advantage in within-subject designs, similarly to between-subject experiments; however, the size of this advantage is difficult to estimate. Further simulations need to be conducted to obtain estimates of power and Type I rates in within-subject designs.

Next, we could only carry out simulations based on a limited selection of datasets. Thus, in the context of sentence processing (Studies 1–3), we only considered data from two-picture VWP experiments, although we have no reason to expect that our conclusions would be substantially affected by the number of pictures in the visual display. More importantly, we only considered a limited selection of *effect types*. As seen from the comparison between Datasets A, C, and D, the type of effect of interest in the eye-tracking response may have a significant impact on the power of the latency comparison tests, with more variability in the eye tracking data leading to lower power. For experiments involving sentence processing, the sample size estimates from Study 1 can be taken as a lower bound on the required sample sizes. In practice, when studying more subtle effects subject to higher variability, as in Study 3, researchers should aim for larger samples. For cohort effects in word recognition tasks, estimates from Study 4 can be used as guidance.

Finally, there are aspects of the latency comparison procedures that may impact their efficacy but which we did not investigate. These include, first and foremost, the choice of statistical test that is performed at each time bin during latency estimation, the choice of effect size threshold and the number of consecutive time bins counted, and the choice of time bin size. We expect that the last parameter may have an impact on the power of the procedures to detect smaller latency differences, especially in the context of low overall variability as in word recognition tasks. We leave this issue for future research.

Another factor which we have not addressed is the number of test items used in the experiment.

Summing up, our study offers a simulation-based validation for the application of resampling-based methods to between-group latency comparison in VWP eye-tracking experiments. We hope that the obtained results will provide useful guidance to researchers in selecting appropriate sample sizes and analysis procedures, including the choice of latency measures, tests, and resampling methods.

## Conclusion

To conclude, we offer some recommendations on the use of non-parametric resampling-based procedures to compare group latencies in VWP experiments:

- *Effect size*. In the context of sentence processing tasks, resampling-based tests are effective if the expected difference in effect latencies is at least 200–300 ms. The power of the tests drops sharply for smaller effects. For word recognition, the tests can reliably detect latency differences as small as 100 ms.
- *Group sizes*. For effects during sentence processing investigated using infrared eye tracking, we recommend samples of at least 36 participants per group to test for 300-ms latency differences and at least 24 participants per group to test for 400-ms differences. For webcam-based eye tracking, the samples should be larger: at least 48 participants per group to test for 300-ms latency differences, and over 36 participants per group to test for 400-ms differences. For cohort effects in word recognition tasks, 25–35 participants per group are needed to reliably detect 100-ms latency differences, and 15 participants per group for 200-ms differences.
- *Choice of test*. The permutation test and percentile bootstrap CIs exhibited the highest power to detect true latency differences across most conditions while maintaining a Type I error rate below the nominal level. Percentile bootstrap CIs performed better in the word recognition paradigm (Study 4), while the permutation test worked better when applied to predictive processing effects (Study 3). When the permutation test is used, caution should be taken to ensure that the exchangeability assumption is justified under the tested null hypothesis. When estimates of uncertainty are required, for example, for latencies within groups or for the difference in latencies between groups, or if there is doubt that the exchangeability assumption is met, percentile bootstrap CIs should be preferred.
- *Choice of latency measure.* Under most conditions, highest power was achieved when using latency measures based on significance in consecutive time bins with an effect size threshold. In fact, across all the studies, applying an effect size threshold conveyed a considerable power advantage. For the test to remain reliable, however, it is essential that the minimally relevant effect size is determined before data analysis is conducted, and ideally given a theoretical justification.
- *Choice of resampling procedure*. We recommend resampling data at the participant level (i.e., *by participant*) in both the permutation and bootstrapping analyses, preserving the internal structure of the original dataset. Bootstrapping *within participants* underestimates uncertainty due to cross-speaker variation, increasing the chances of a false-positive result.

A template R script implementing both the permutation- and bootstrap-based latency comparison procedures can be accessed at https://osf.io/9dpgn/overview.

## Supplementary Information

Below is the link to the electronic supplementary material.

Supplementary file1 (DOCX 22604 KB)

## Data Availability

All the seed datasets and full results are available at the following link: https://osf.io/9dpgn/overview.

## Footnotes

1 Stone et al. (2021) report a sample of 70 participants in Experiment 2, but we found that one of the participants lacked any data points in the match condition within the ROI. This participant was excluded from Dataset C.

2 ABC CIs provide an approximation of bias-corrected accelerated (BCa) CIs, another advanced method for calculating bootstrap confidence intervals (Efron, 1987). Unfortunately, calculating BCa CIs is computationally costly, and thus could not be implemented in the simulation study due to practical limitations.

3 The distinct “populations” may be predefined or created as part of the experiment, e.g., in a randomized between-subject design.

4 The by-participant bootstrap described above can also be considered a form of stratified bootstrap since data are resampled within groups. To avoid confusion, we will continue to use the term *stratified bootstrap* to refer to resampling within participants and time bins, and use the term *by-participant bootstrap* for resampling by participant within groups.

5 In Study 2, 99.9% of the proportions were 0 or 1 already prior to binarizations. In Study 3 it was 100% due to the fact that that the saccades necessary for switching between the pictures took longer than 40 ms, and all saccade data were excluded from the dataset.

6 The permutation test tended to perform worse than percentile CIs with the smallest group size (12 participants per group), but neither of the tests approached 80% for any effect size with such small groups.

## Code availability

All the scripts for the simulations and analysis are available at the following link: https://osf.io/9dpgn/overview.
