# Supplementary material for: Comparing effect latencies in the visual world paradigm: Monte Carlo simulations to assess resampling-based procedures
Source: Behav Res Methods. 2026 Feb 23;58(3):70. doi: 10.3758/s13428-025-02934-6 (PMC12929238; doi:10.3758/s13428-025-02934-6)
Supplement: Supplementary file 1 — Supplementary file1 (DOCX 22604 KB) [file 13428_2025_2934_MOESM1_ESM.docx]

**Appendix A. Full results**


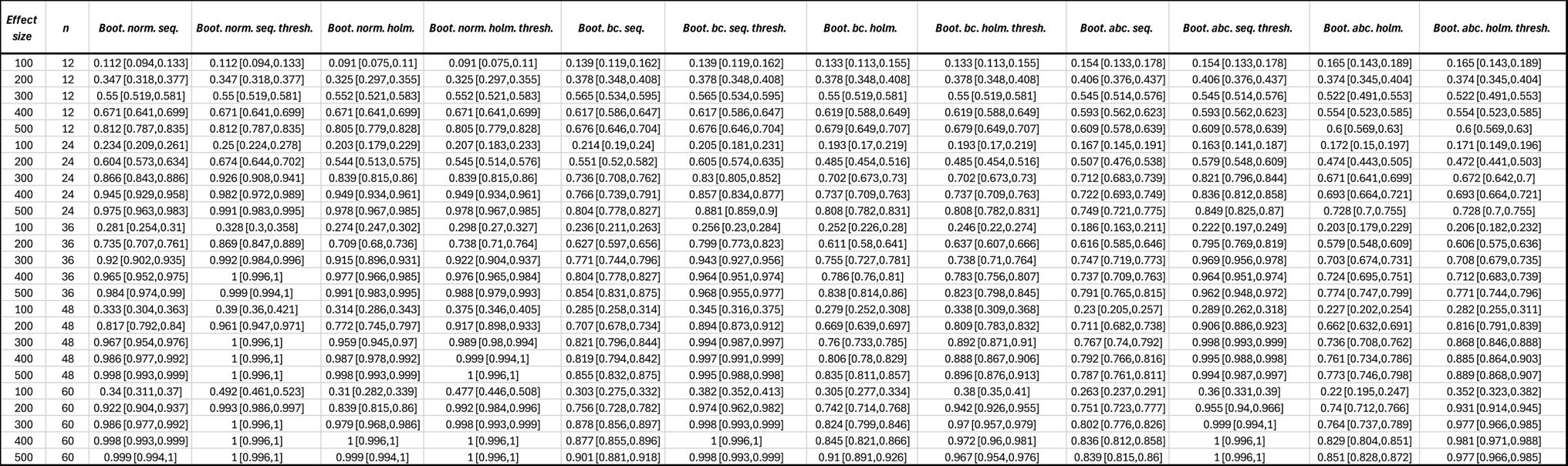

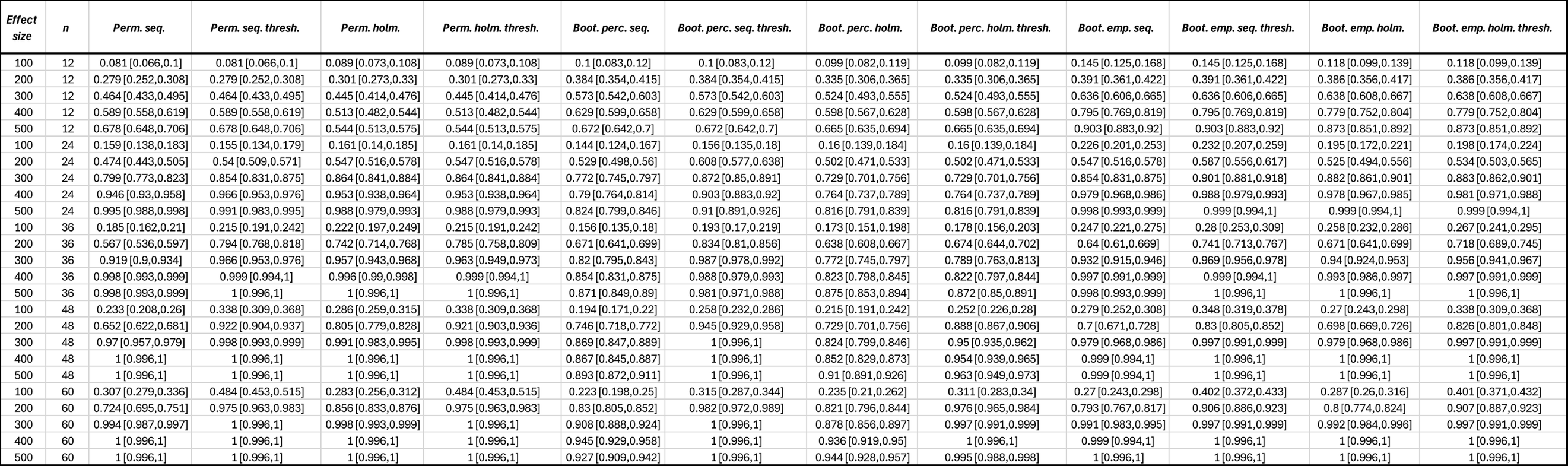


Table 1. Study 1: Power estimates for all combinations of effect size, group size (*n*) and procedure parameters, with 95% Wilson CIs.


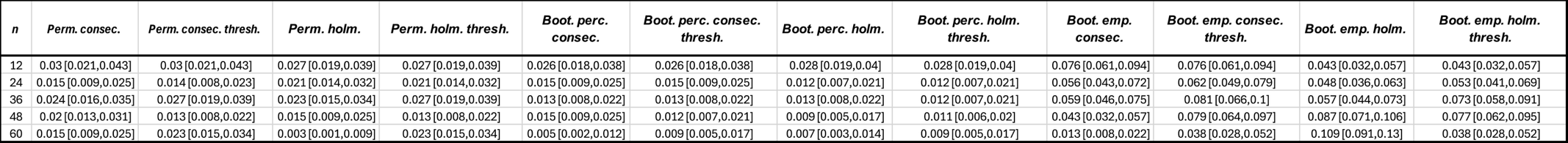

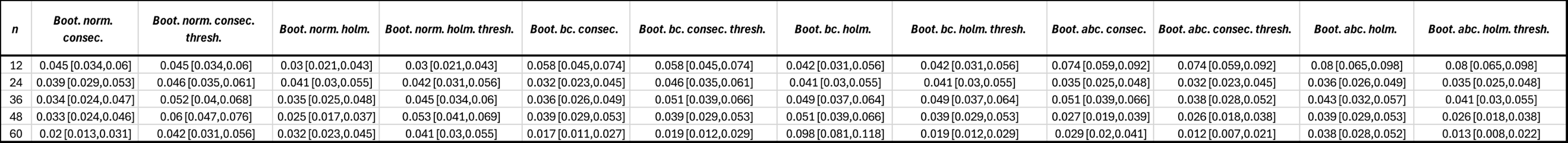


Table 2. Study 1: Estimated Type I error rates for all parameters of the analysis procedures and group sizes (*n*), with 95% Wilson CIs.

Table 3a. Study 2: Power estimates for all combinations of effect size, group size (*n*) and procedure parameters, with 95% Wilson CIs (first half).

Table 3b. Study 2: Power estimates for all combinations of effect size, group size (*n*) and procedure parameters, with 95% Wilson CIs (second half).

Table 4. Study 2: Estimated Type I error rates for all parameters of the analysis procedures and group sizes (*n*), with 95% Wilson CIs.

Table 5. Study 3: Power estimates for all combinations of effect size, group size (*n*) and procedure parameters, with 95% Wilson CIs.

Table 6. Study 3: Estimated Type I error rates for all parameters of the analysis procedures and group sizes (*n*), with 95% Wilson CIs.


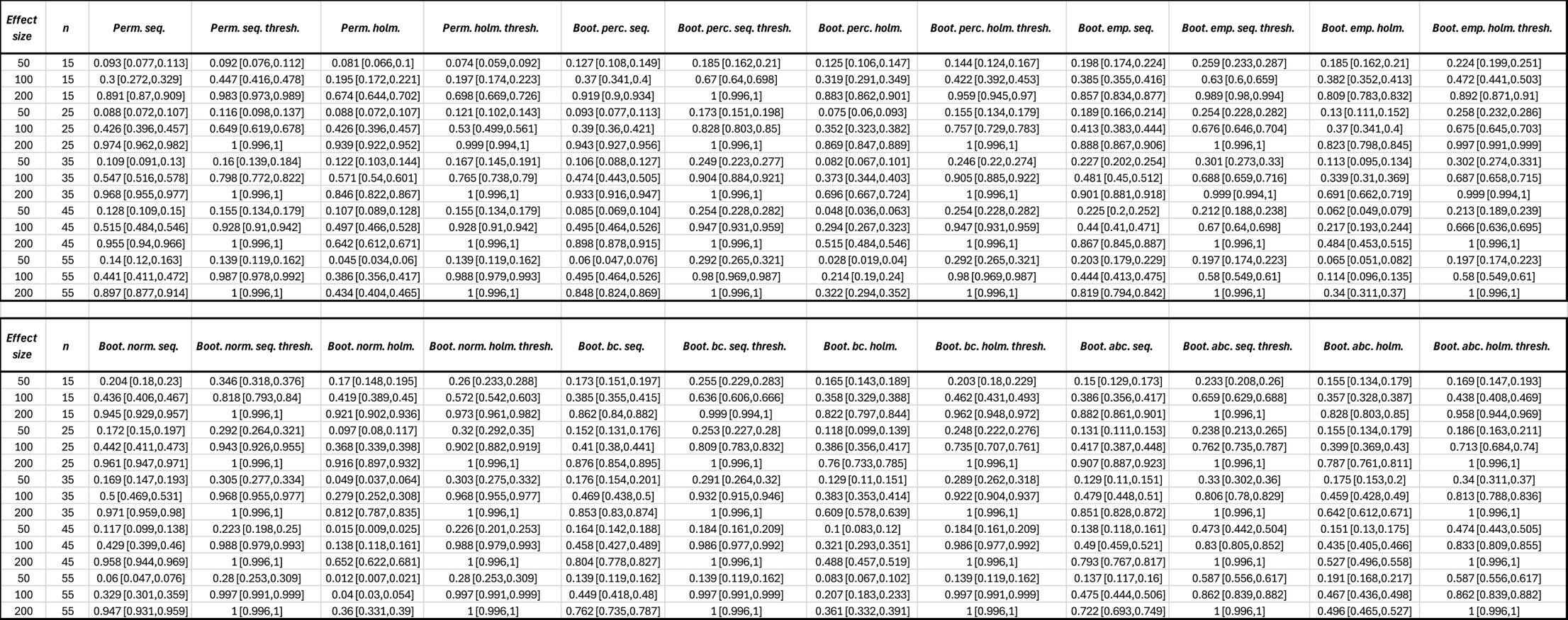

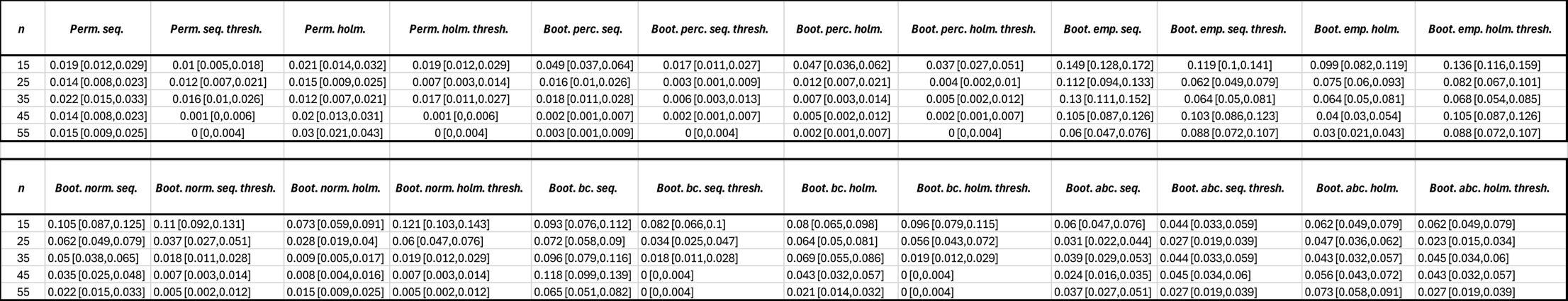


Table 7. Study 4: Power estimates for all combinations of effect size, group size (*n*) and procedure parameters, with 95% Wilson CIs.

Table 8. Study 4: Estimated Type I error rates for all parameters of the analysis procedures and group sizes (*n*), with 95% Wilson CIs.

*
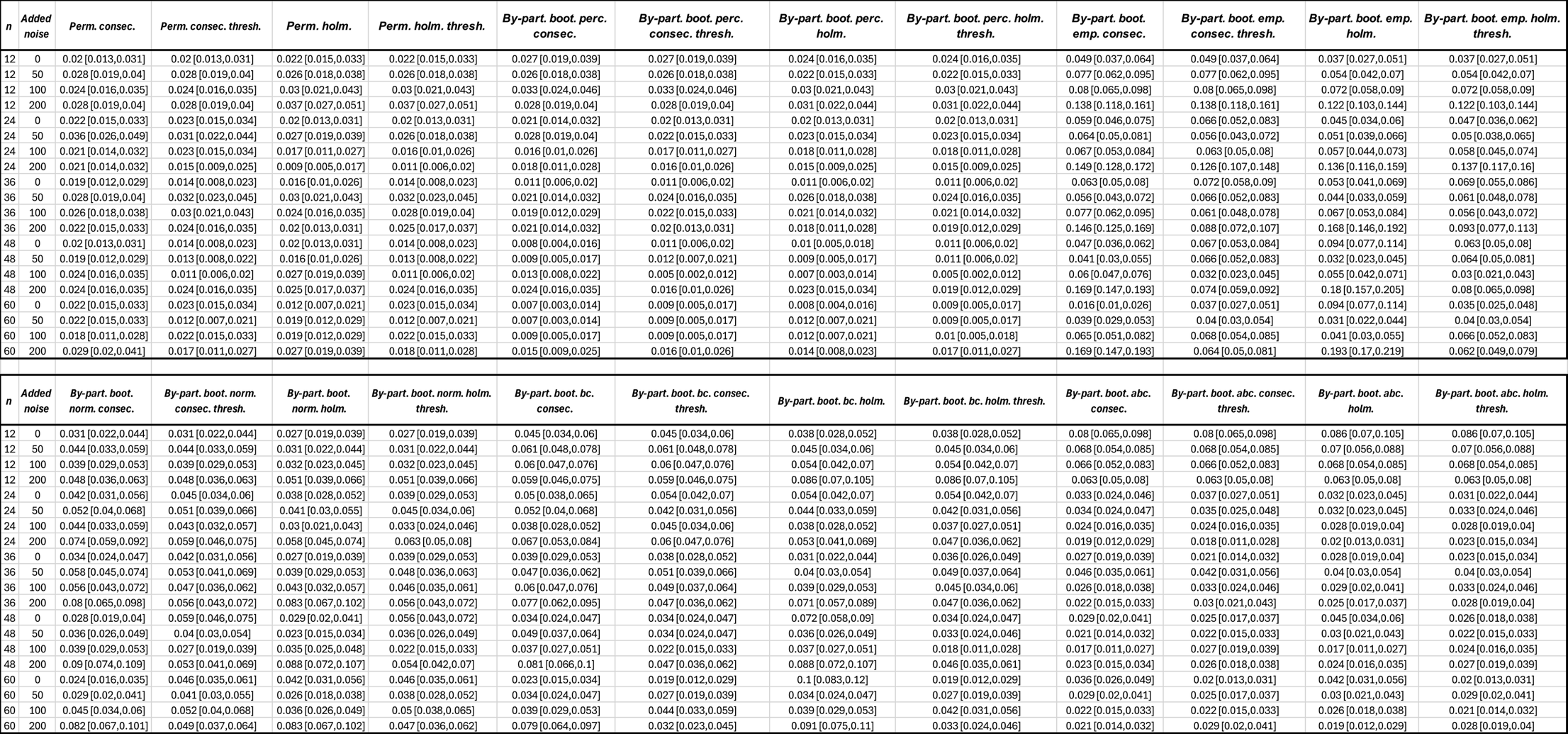
*

Table 9a. Study 4a: Estimated Type I error rates for the permutation and by-participant bootstrap procedures, by group size (*n*) and added noise level, with 95% Wilson CIs.

*
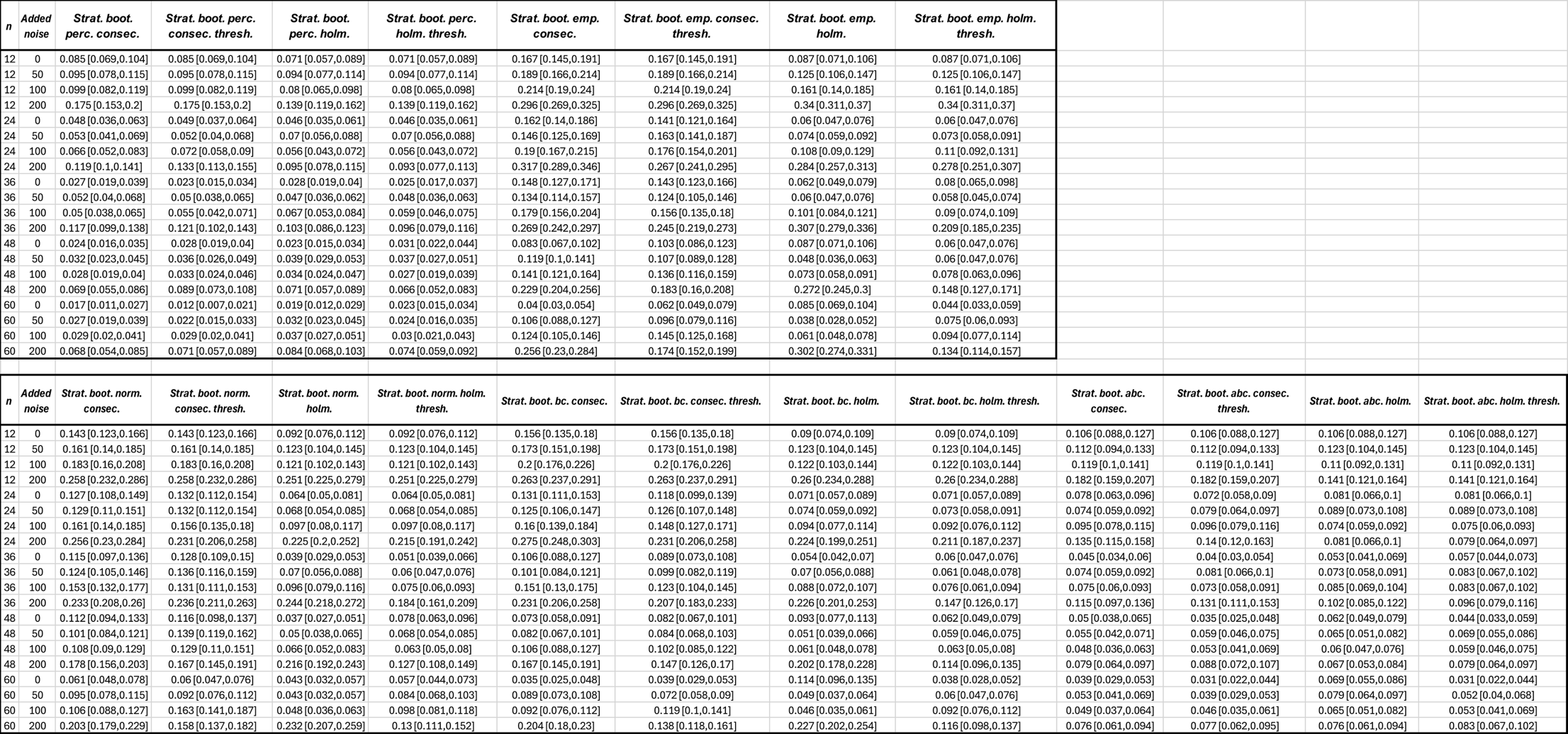
*

Table 9b. Study 4a: Estimated Type I error rates for the stratified bootstrap procedure, by group size (*n*) and added noise level, with 95% Wilson CIs.

*
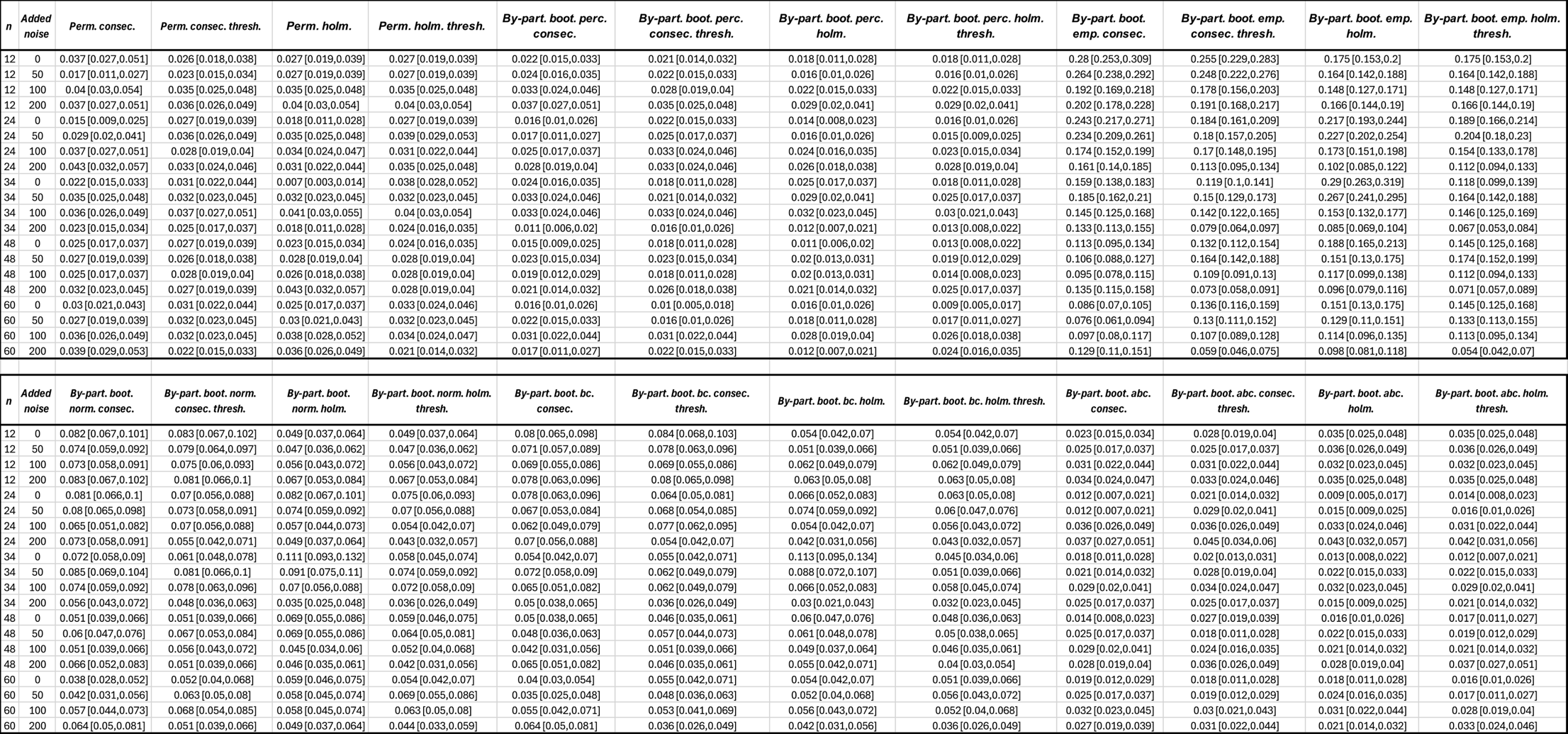
*

Table 10a. Study 4b: Estimated Type I error rates for the permutation and by-participant bootstrap procedures, by group size (*n*) and added noise level, with 95% Wilson CIs.

*
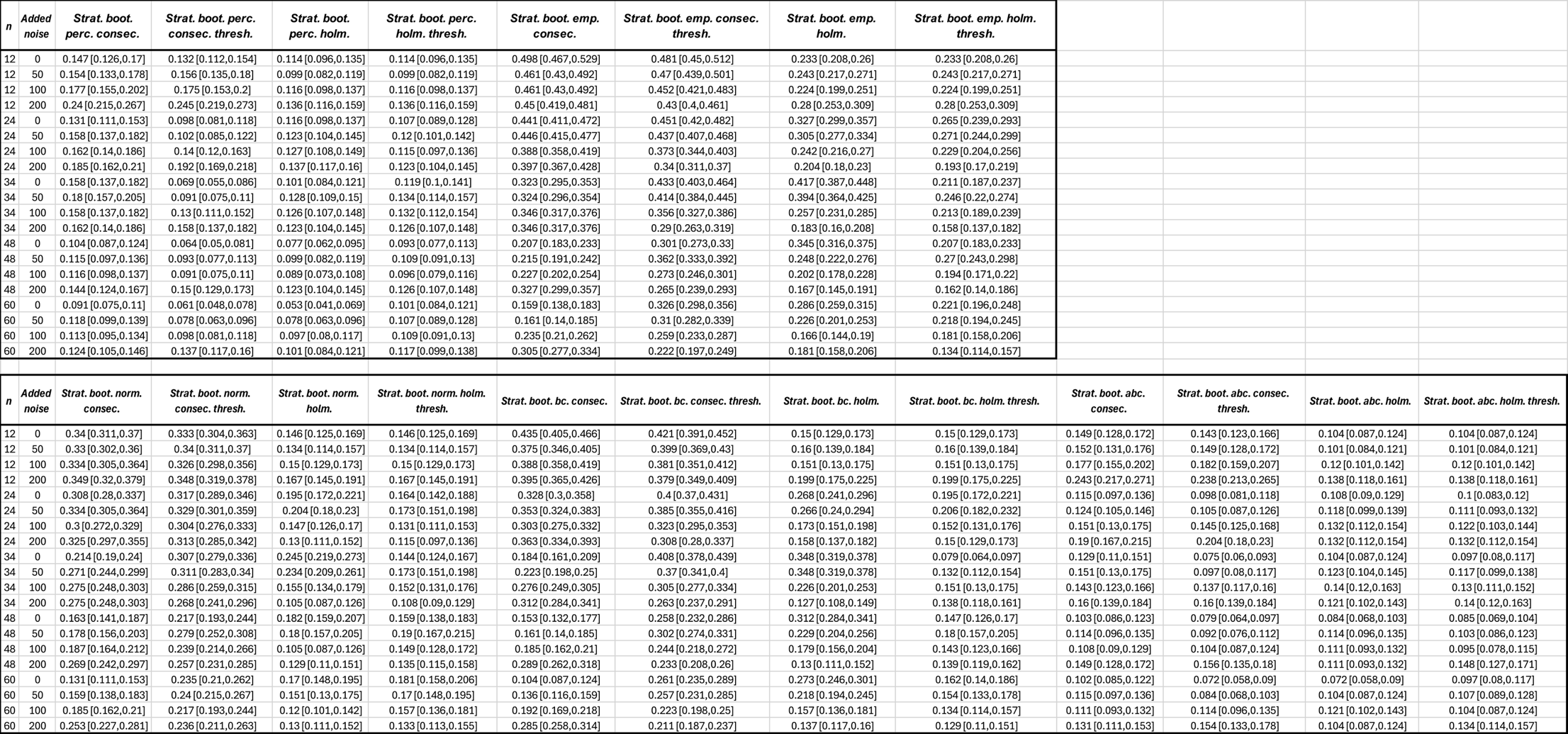
*

Table 10b. Study 4b: Estimated Type I error rates for the stratified bootstrap procedure, by group size (*n*) and added noise level, with 95% Wilson CIs.

**Appendix B. Pooling data for more precise estimation of power and false-positive rates for bootstrap-based tests**

The following equivalence holds for all the tested latency measures:

1. $L\left( D+n \right)= L(D)+n$

where *D* is an eye tracking dataset, $D+n$ denotes the dataset resulting from adding *n* to every time index in *D*, and *L*(*D*) is the latency measure calculated from *D.* Consequently, for any bootstrap iteration *i*:

1. $d_{n}^{*i}=L\left( S^{*i}+n \right)- L\left( B^{*i} \right)= L\left( S^{*i} \right)- L\left( B^{*i} \right)+n$

where $B^{*i}$ and $S^{*i}$ are the resampled eye tracking data for the Baseline and Shifted groups, respectively, *n* is the true effect size, and $d_{n}^{*i}$is the difference in latency measures between the sampled groups.

This implies that the bootstrap distribution of latency differences for an effect size *n* can be used to derive the latency difference distribution for any other effect size *m*. Specifically, for any bootstrap iteration *i*:

1. $d_{m}^{*i}=L\left( S^{*i} \right)- L\left( B^{*i} \right)+m= L\left( S^{*i} \right)- L\left( B^{*i} \right)+n+\left( m-n \right)=d_{n}^{*i}+(m-n)$

Thus, for any group size, all 6000 simulations across the six tested effect sizes can be pooled to calculate more precise estimates of power and false positive rates of the bootstrap confidence intervals over a broader range of effect sizes.^[[1]](#footnote-1)^ The results are presented in Figs. B1-B9.

**Fig. B1.** Study 1: Power estimates based on pooled data for different types of bootstrap CIs, by group and effect size. Latency measure based on effect in consecutive time bins.


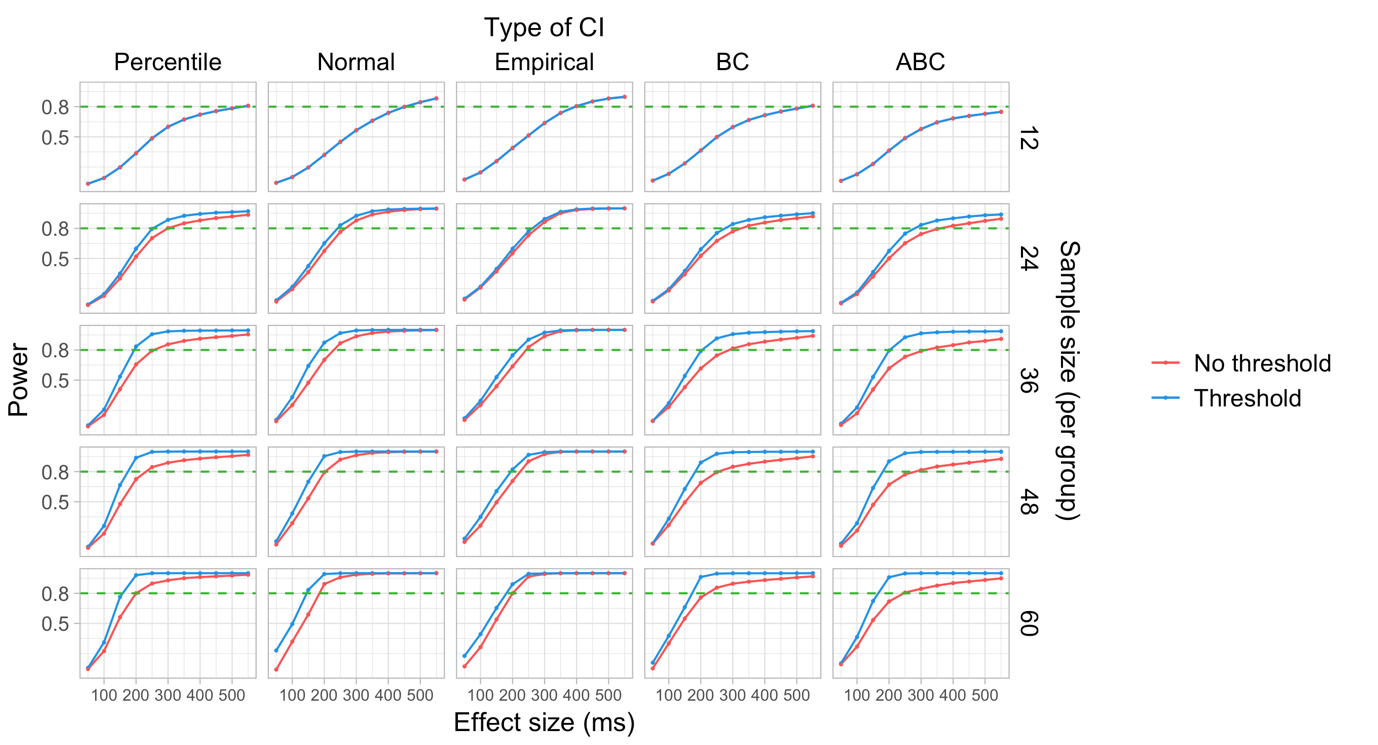


**Fig. B2.** Study 1: Power estimates based on pooled data for different types of bootstrap CIs, by group and effect size. Latency measure based on earliest effect after Holm-Bonferroni correction.


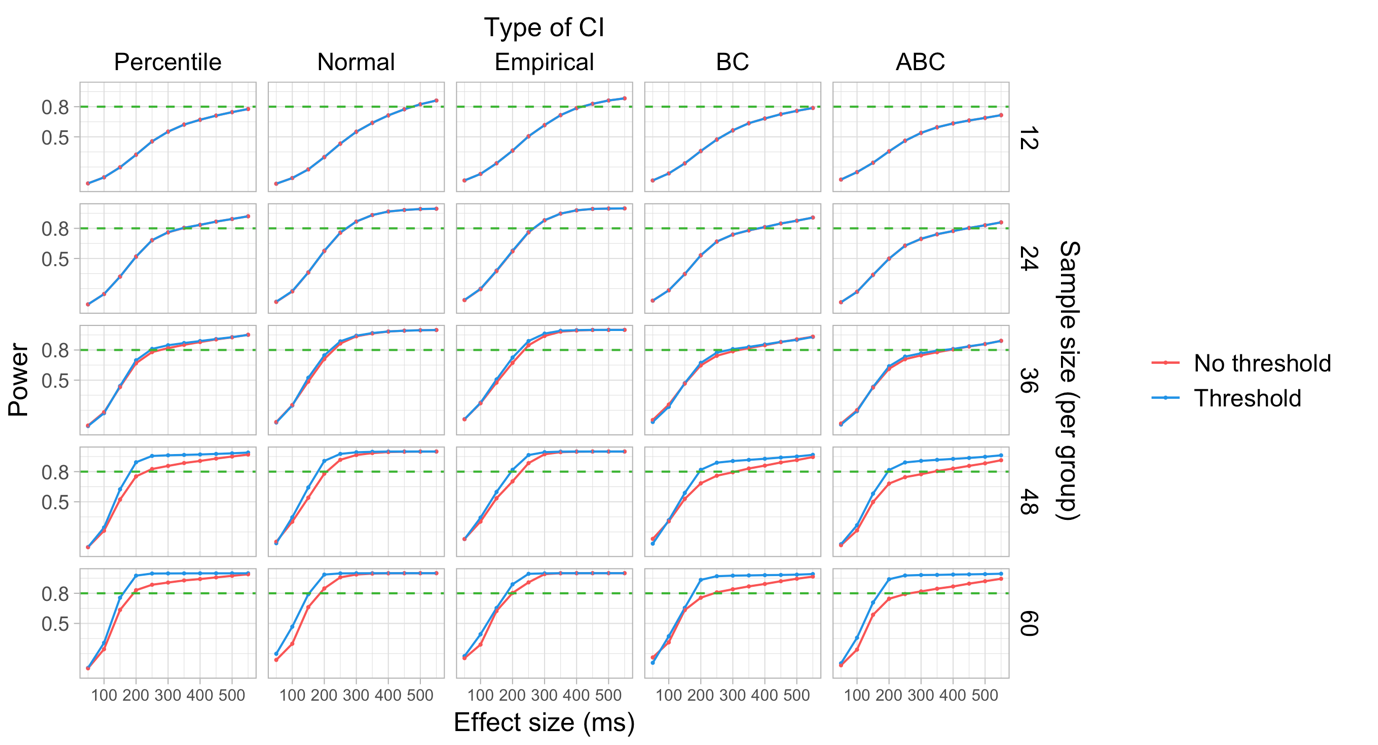


**Fig. B3.** Study 1: False positive rates based on pooled data for different types of bootstrap CIs, by latency measure and sample size.


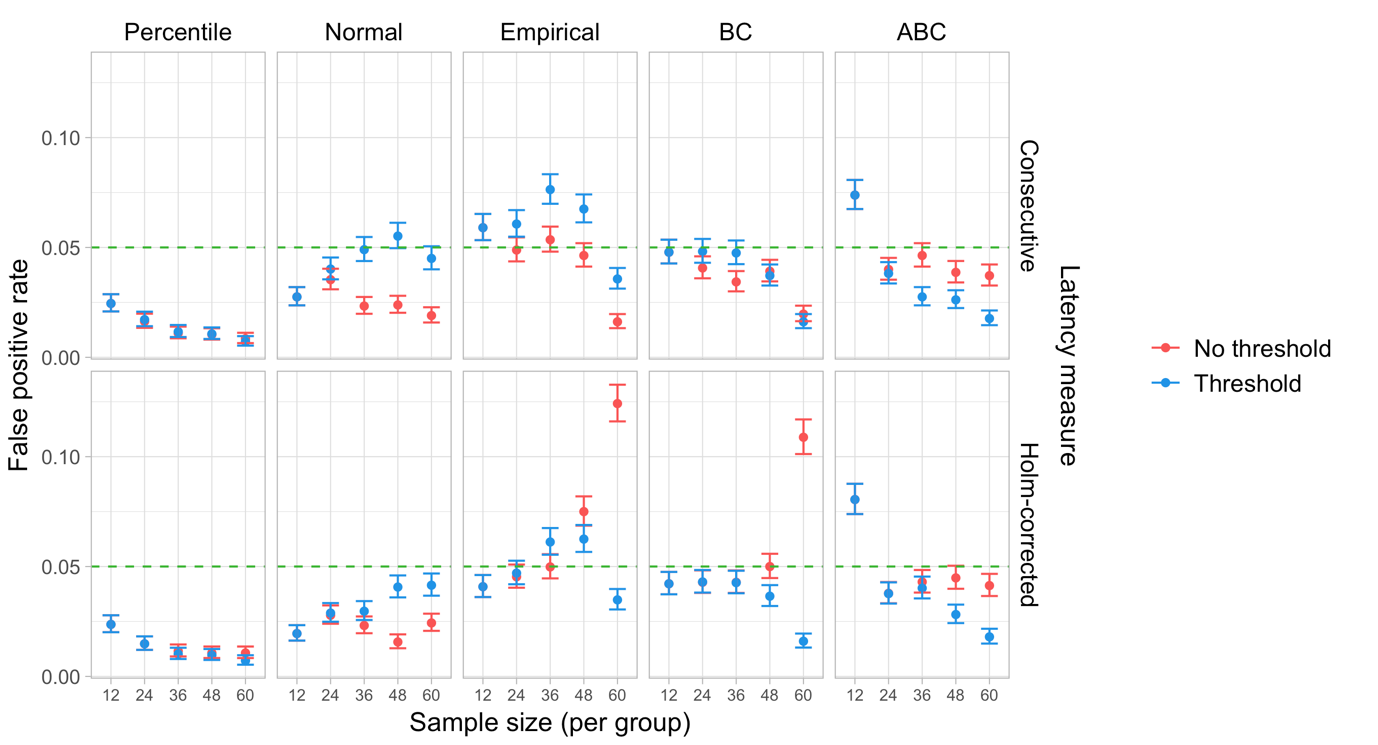

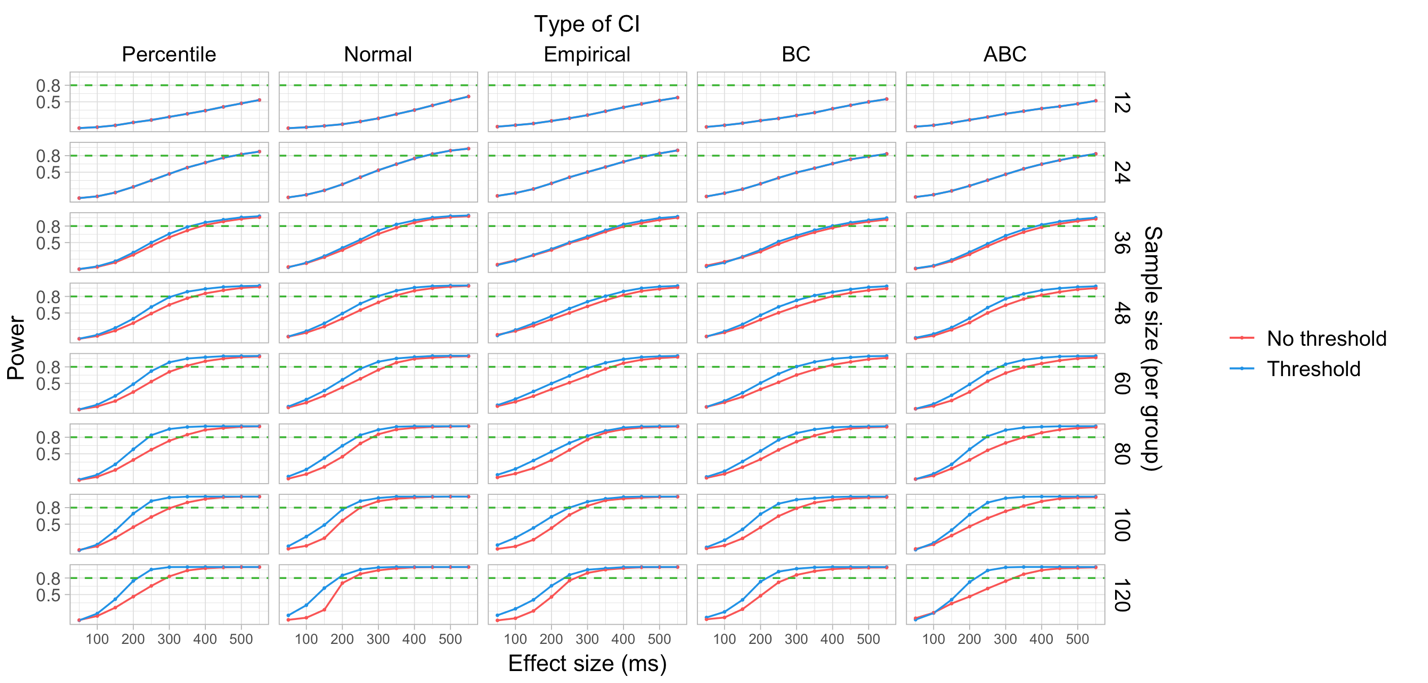


**Fig. B4.** Study 2: Power estimates based on pooled data for different types of bootstrap CIs, by group and effect size. Latency measure based on effect in consecutive time bins.


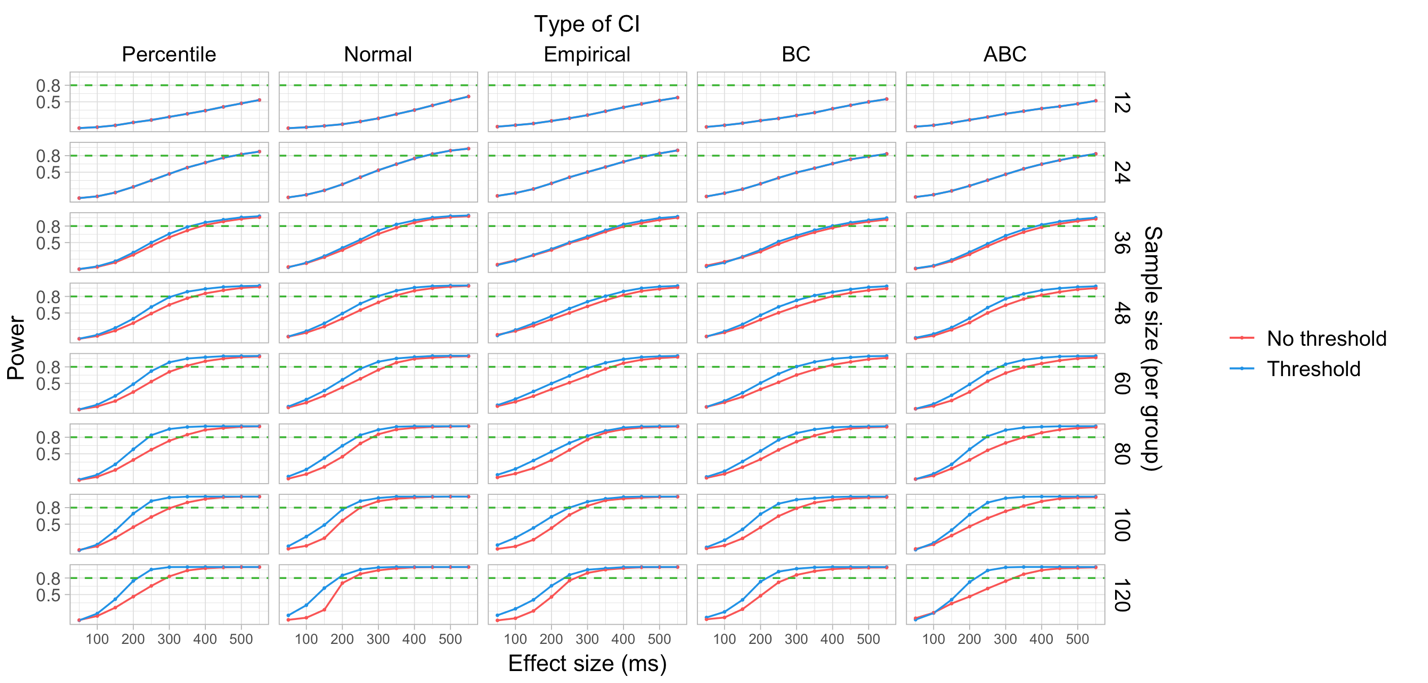


**Fig. B4.** Study 2: Power estimates based on pooled data for different types of bootstrap CIs, by group and effect size. Latency measure based on effect in consecutive time bins.


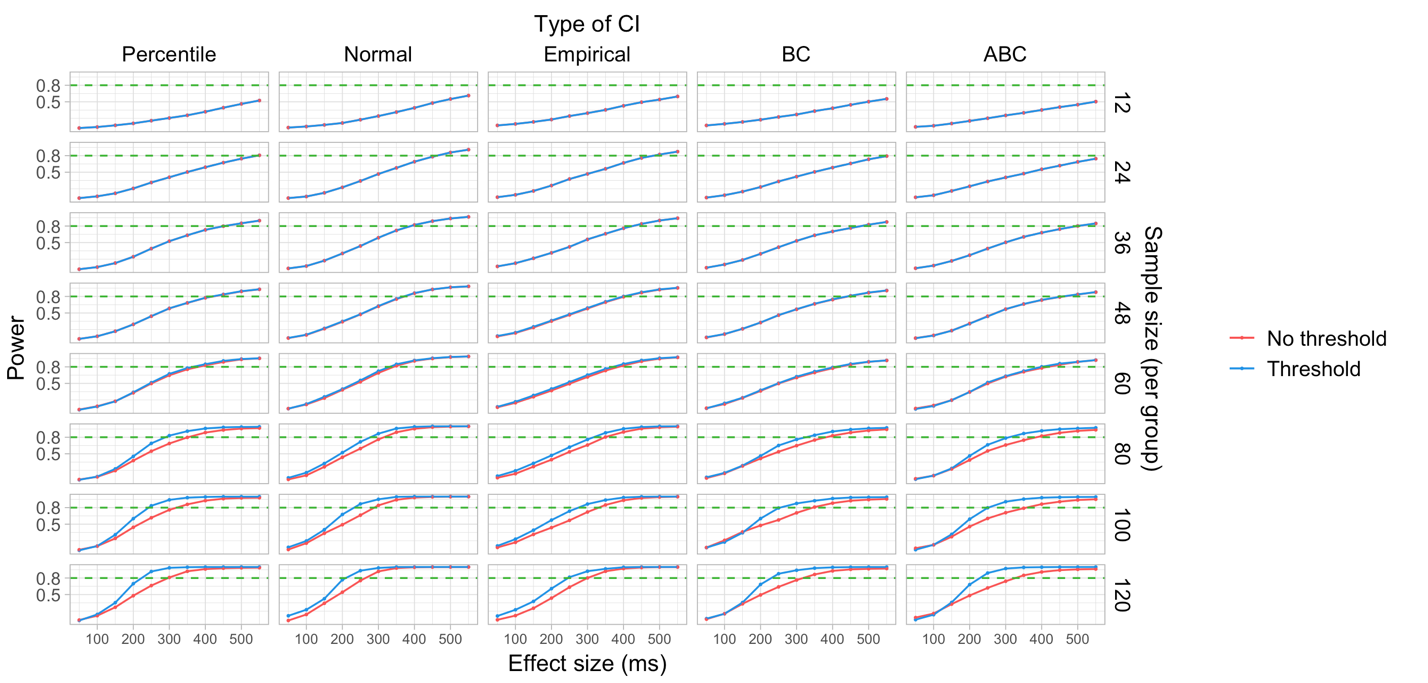


**Fig. B5.** Study 2: Power estimates based on pooled data for different types of bootstrap CIs, by group and effect size. Latency measure based on earliest effect after Holm-Bonferroni correction.

**Fig. B6.** Study 2: False positive rates based on pooled data for different types of bootstrap CIs, by latency measure and sample size.


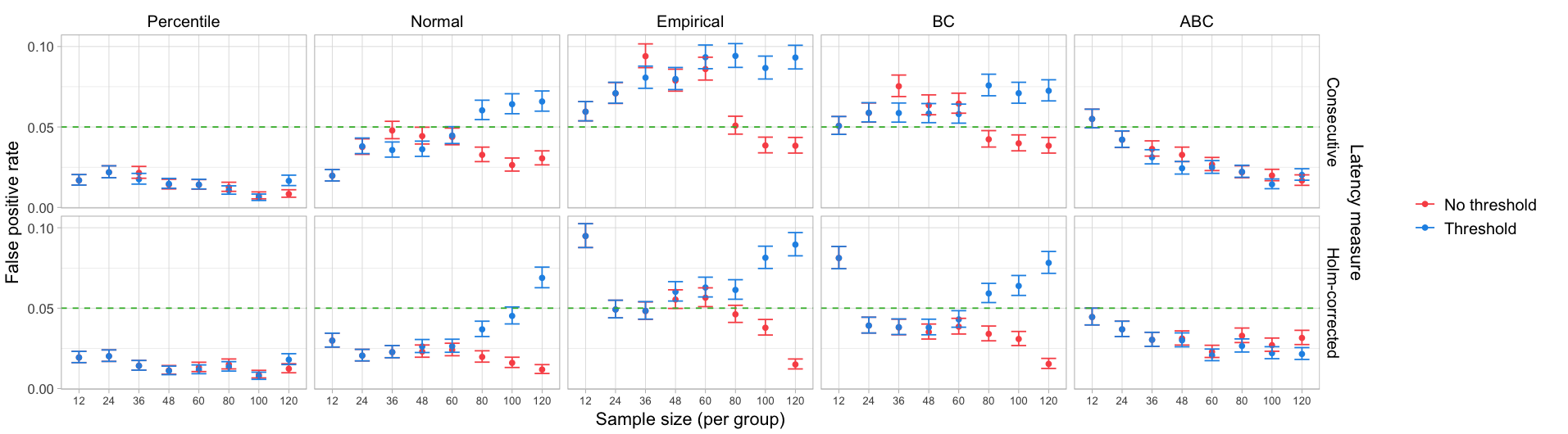


**Fig. B8.** Study 3: Power estimates based on pooled data for different types of bootstrap CIs, by group and effect size. Latency measure based on earliest effect after Holm-Bonferroni correction.


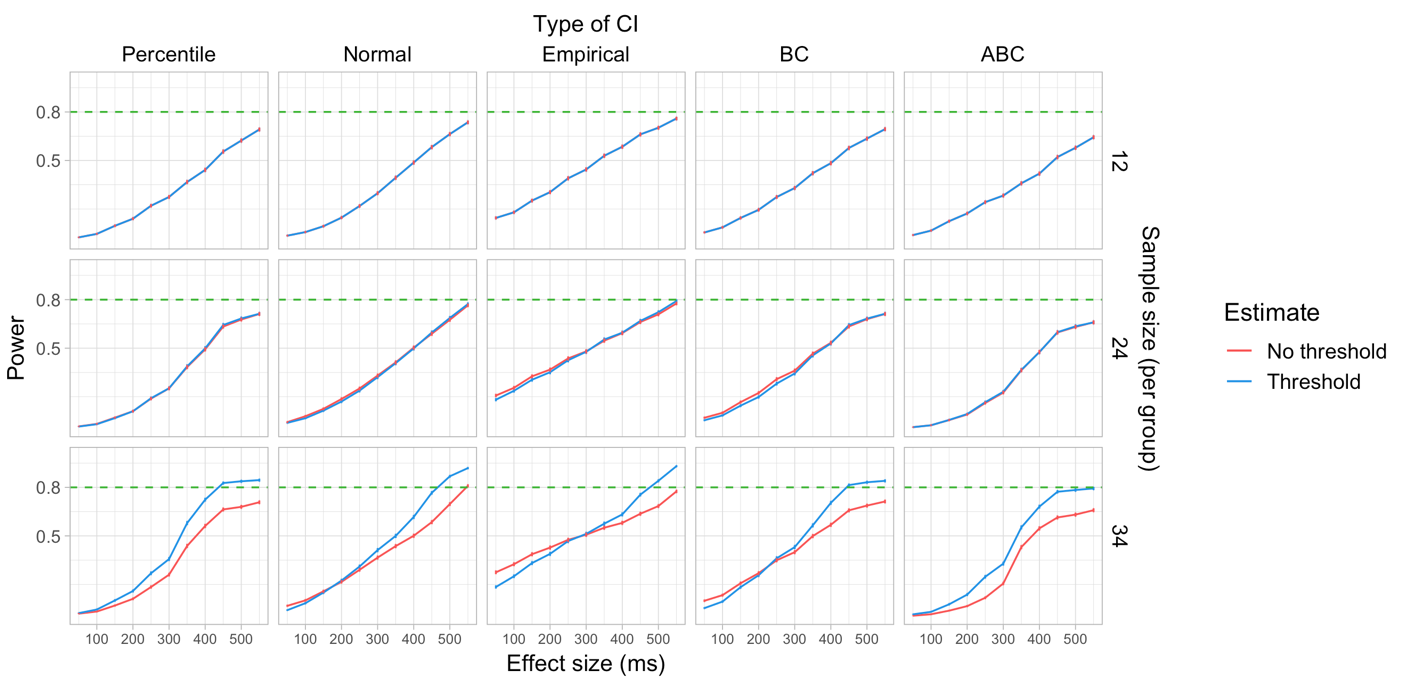


**Fig. B7.** Study 3: Power estimates based on pooled data for different types of bootstrap CIs, by group and effect size. Latency measure based on effect in consecutive time bins.


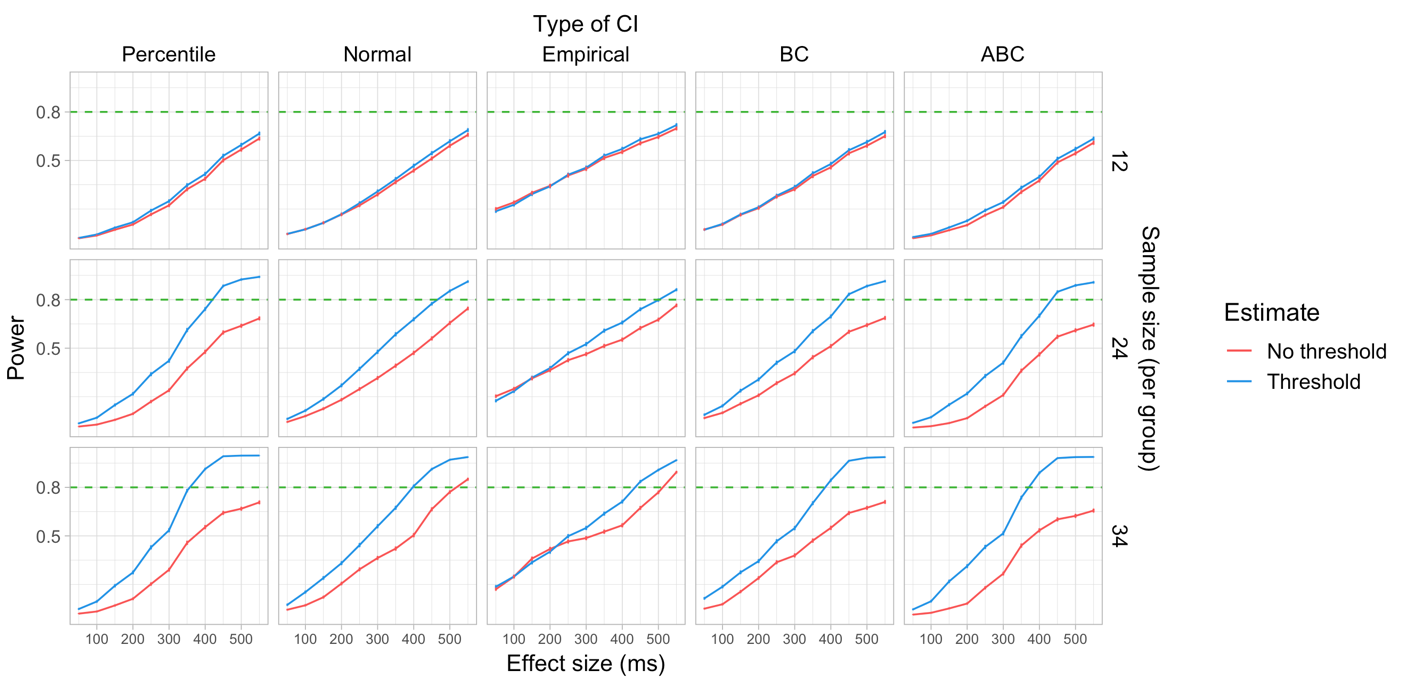

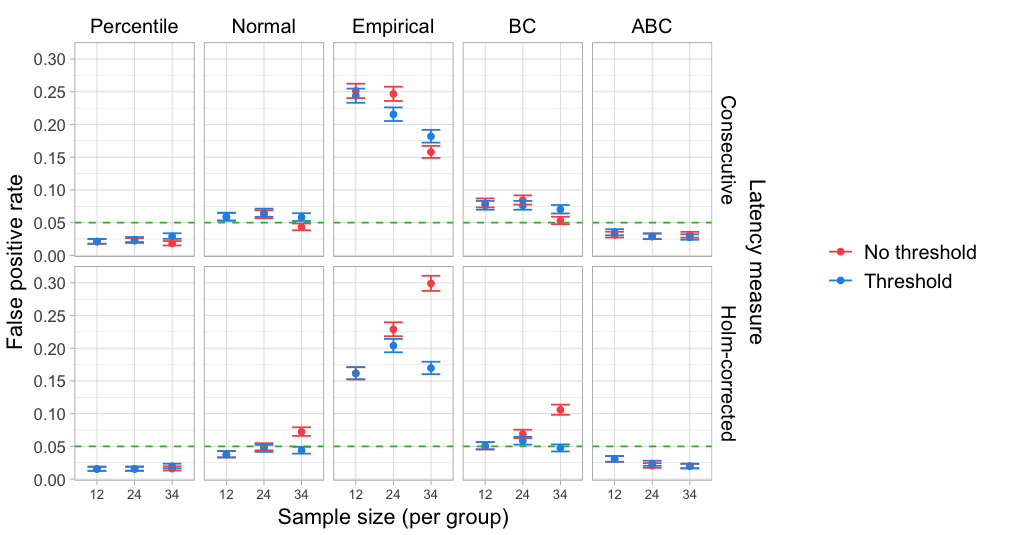


**Fig. B9.** Study 3: False positive rates based on pooled data for different types of bootstrap CIs, by latency measure and sample size.

1. Note that such pooling is not possible for the permutation test. Take a simulation *j* for effect size  *n*, such that *B_j_* and (*S_j_+n*) are the datasets corresponding to the sampled Baseline and Shifted groups, respectively. On each permutation *i* latencies *L* are computed for random reshuffles of datasets *B_j_* and (*S_j_+n*). Crucially, we cannot use these latencies to derive *L* for reshuffled datasets *B_j_* and (*S_j_+m*) for a different effect size *m*. [↑](#footnote-ref-1)
